# Clinical significance in pediatric oncology randomized controlled treatment trials: a systematic review

**DOI:** 10.1186/s13063-018-2925-8

**Published:** 2018-10-05

**Authors:** A. Fuchsia Howard, Karen Goddard, Shahrad Rod Rassekh, Osama A Samargandi, Haroon Hasan

**Affiliations:** 1 School of Nursing, The University of British Columbia, T201-2211 Wesbrook Mall, Vancouver, BC V6T 2B5 Canada; 2 Department of Radiation Oncology, BC Cancer, Vancouver, BC V5Z 4E6 Canada; 3 Division of Hematology/Oncology, BC Children’s Hospital, Vancouver, BC V6H 3N1 Canada; 4 Division of Plastic Surgery, QEII Health Sciences Centre, Halifax, NS B3H 3A7 Canada; 5 Epi Methods Consulting, Toronto, ON M5V 0C4 Canada

**Keywords:** Clinical significance, Minimally clinically important difference, Randomized controlled trials, Pediatric oncology

## Abstract

**Background:**

Clinical significance in a randomized controlled trial (RCT) can be determined using the minimal clinically important difference (MCID), which should inform the delta value used to determine sample size. The primary objective was to assess clinical significance in the pediatric oncology randomized controlled trial (RCT) treatment literature by evaluating: (1) the relationship between the treatment effect and the delta value as reported in the sample size calculation, and (2) the concordance between statistical and clinical significance. The secondary objective was to evaluate the reporting of methodological attributes related to clinical significance.

**Methods:**

RCTs of pediatric cancer treatments, where a sample size calculation with a delta value was reported or could be calculated, were systematically reviewed. MEDLINE, EMBASE, and the Cochrane Childhood Cancer Group Specialized Register through CENTRAL were searched from inception to July 2016.

**Results:**

RCTs (77 overall; 11 and 66), representing 95 (13 and 82) randomized questions were included for non-inferiority and superiority RCTs (herein, respectively). The minority (22.1% overall; 76.9 and 13.4%) of randomized questions reported conclusions based on clinical significance, and only 4.2% (15.4 and 2.4%) explicitly based the delta value on the MCID. Over half (67.4% overall; 92.3 and 63.4%) reported a confidence interval or standard error for the primary outcome experimental and control values and 12.6% (46.2 and 7.3%) reported the treatment effect, respectively. Of the 47 randomized questions in superiority trials that reported statistically non-significant findings, 25.5% were possibly clinically significant. Of the 24 randomized questions in superiority trials that were statistically significant, only 8.3% were definitely clinically significant.

**Conclusions:**

A minority of RCTs in the pediatric oncology literature reported methodological attributes related to clinical significance and a notable portion of statistically insignificant studies were possibly clinically significance.

**Electronic supplementary material:**

The online version of this article (10.1186/s13063-018-2925-8) contains supplementary material, which is available to authorized users.

## Background

Cancer among children is rare, accounting for less than 1% of all new cases in Canada [1]. Over the past 50 years, the 5-year relative survival rate for pediatric cancers has risen dramatically, from 10 to 83% [2, 3], largely because of treatment advances and high rates of clinical trials participation, estimated to be upwards of 60% [4]. Pediatric clinical trials are remarkably complex because of the lower incidence of disease, safety concerns, stringent regulatory requirements and limited commercial interest [5]. As such, randomized controlled trials (RCTs) are predominately multi-institutional, resource intensive, often take years to complete and rarely have pharmaceutical industry support [6]. By and large, national and international collaborative efforts, such as the Children’s Oncology Group, coordinate the majority of trials, the results of which often provide the basis for changes in treatment regimens and standard of care [7]. Even one trial can dramatically change standard of care [8, 9].

The concept of clinical significance is now considered crucial in RCT planning and interpretation [10]. The 2010 Consolidated Standards of Reporting Trials (CONSORT) Statement acknowledged the importance of assessing study results based on sample size calculations and assumptions that include clinical significance [11]. Clinical significance can be determined using the minimal clinically important difference (MCID); “*the smallest treatment effect that would result in a change in patient management, given its side effects, costs, and inconveniences*” [12, 13]. It is critical that a study be powered based on a delta value that reflects the MCID. The delta value component of the sample size calculation is the difference between the experimental and the control group that can be detected based on the type 1 (*α* value) and type 2 (*β* value) errors. A delta value that reflects the MCID ensures that the sample size calculation allows for an evaluation of clinical significance. In addition, the appropriate clinical interpretation of results requires authors to report methodological attributes related to clinical significance, such as justification of the MCID [11, 14, 15]. Thus, it is essential for studies to be designed and interpreted based on an evidence-based MCID and clinically relevant measures, such as confidence intervals (CIs), which provide information on statistical significance, and the direction and size of the treatment effect [12, 16–18].

Research suggests that RCT authors rely primarily on statistical significance, they do not consistently provide their own interpretation of the clinical importance of results, and they rarely provide sufficient information to enable readers to draw their own conclusions [13, 19, 20]. The degree to which clinical significance has been assessed in the pediatric oncology literature remains unknown. The primary study objective was to assess clinical significance, and reporting of clinical significance, in the pediatric oncology RCT literature by, first, evaluating the relationship between the treatment effect (with its associated CI) and the delta value, as reported in the sample size calculation and, second, assessing the concordance between statistical and clinical significance. The secondary study objective was to assess methodological attributes related to clinical significance.

## Methods

This systematic review was conducted following a pre-defined protocol, which was informed by the Preferred Reporting Items for Systematic Reviews and Meta-Analyses (PRISMA) Statement [21].

### Search strategy

The academic literature was systematically searched using a comprehensive search strategy to identify all RCTs in pediatric oncology (Additional file 1: Appendix A). We searched MEDLINE, EMBASE, and the Cochrane Childhood Cancer Group Specialized Register through CENTRAL from inception to July 2016. Our search strategy was developed by initially using the Canadian Agency for Drugs and Technologies in Health search filter to identify RCTs [22] and subsequently adapted to identify RCTs in pediatric oncology using the Cochrane Childhood Cancer Search Filter, which has been validated by Leclercq et al. [23]. We also assessed the reference lists of the studies that fulfilled our inclusion criteria to identify additional studies. Our search was restricted to studies in English and was inclusive of the published literature.

### Eligibility criteria

Studies were deemed eligible if the study adhered to a RCT study design (i.e., did not included a non-randomized component or a historical control), where the study population consisted of pediatric patients diagnosed with cancer and the primary outcome of the trial was a relevant cancer treatment outcome (e.g., a treatment regimen assessing overall survival, event-free survival, etc.), and the trial was a phase III trial that did not stop early due to futility. This did not include studies wherein the primary outcome was treatment complications or side effects, pharmacokinetic trials, toxicity trials, non-clinical interventions, or drug safety profile trials. Only studies that reported a sample size calculation where a delta value was reported or could be calculated for the randomized question were included. Trials that were long-term follow-up studies were excluded and only studies that reported the most recent trial results were reported. Only phase III trials that were not stopped early due to futility were eligible for inclusion. RCTs where the study population consisted of both pediatric and adult patients were deemed eligible if adults were less than or equal to 25 years of age. We chose this age range to reflect norms in pediatric oncology treatment research wherein trials most often include participants up to 21 years of age, though many have also included participants up to age 25 years because of the potential benefit for these slightly older patients. Restricting the age limit to 21 years would result in the exclusion of a number of trials where the majority of participants were aged less than 21 years.

### Study identification

Two investigators (HH and KN, non-independently) screened the title and abstracts based on the specified inclusion criteria. The full text was retrieved and reviewed if the title and abstract was insufficient to determine fulfillment of inclusion criteria. Subsequently, one investigator (HH) conducted a full-text review to assess all of the studies that passed through the first round of title and abstract for inclusion eligibility. The principal investigator (AFH) was available to resolve any discrepancies or disagreements encountered during study selection.

### Data extraction

A standardized data extraction template was developed to collect attributes relevant to clinical and statistical significance in addition to general characteristics. The data extraction template was initially piloted on a sample of 15 included studies to ensure that pertinent information was captured and subsequently finalized based on the results of the pilot. Data was collected by one investigator (HH) based on each of the randomized questions within all RCTs, thereby capturing each outcome and the corresponding reported sample size calculation.

### Analysis

#### Characteristics of included studies

The characteristics of the studies included in our systematic review included: journal, region, and year of publication; source of funding; whether the RCT included exclusively children or adults and children; the disease site of focus of the RCT (hematological, central nervous system, non-central nervous system solid tumor); RCT study design (2 × 2 factorial, greater than two arms; parallel-group); trial group; primary outcome defined as time-to-event or dichotomous; primary outcome intervention (chemotherapy, multimodal therapy, hematopoietic stem-cell transplant, radiation therapy). This analysis was based on studies and stratified by RCT type (non-inferiority or superiority).

#### Reporting of methodological attributes associated with clinical significance

The selection of methodological attributes related to clinical significance were informed by evidence from the literature and the expertise of the research team [11, 13, 24, 25]. These attributes consisted of: explicitly identifying the expected magnitude of difference as the MCID and providing justification for why this MCID was selected, whether it be based on clinical relevance or methodological; reporting the delta value as an absolute and/or relative difference stratified by primary outcome type (time-to-event or dichotomous); reporting anticipated control and experimental values for which the delta value was derived from and providing the rationale for why the assumed control value was selected; type 1 error (*α* value) and number of sides of *p* value; power (1 − *β* value); reporting statistical significance of the primary outcome via a *p* value; reporting a confidence interval (CI) or standard error around the experimental and control estimates for the primary outcome; reporting treatment effect (i.e., experimental value – control value); reporting, within the discussion, an assessment of the clinical importance of the results of the primary outcome through an interpretation of the results based on the delta value specified in the sample size calculation. As methodological attributes are relevant to the primary outcome, this analysis was restricted to randomized questions stratified by RCT type (non-inferiority or superiority). Therefore, studies that involved multiple primary outcomes (e.g., 2 × 2 factorial trials, etc.), would have more than one randomized question.

### Clinical significance

**Fig. 1:**
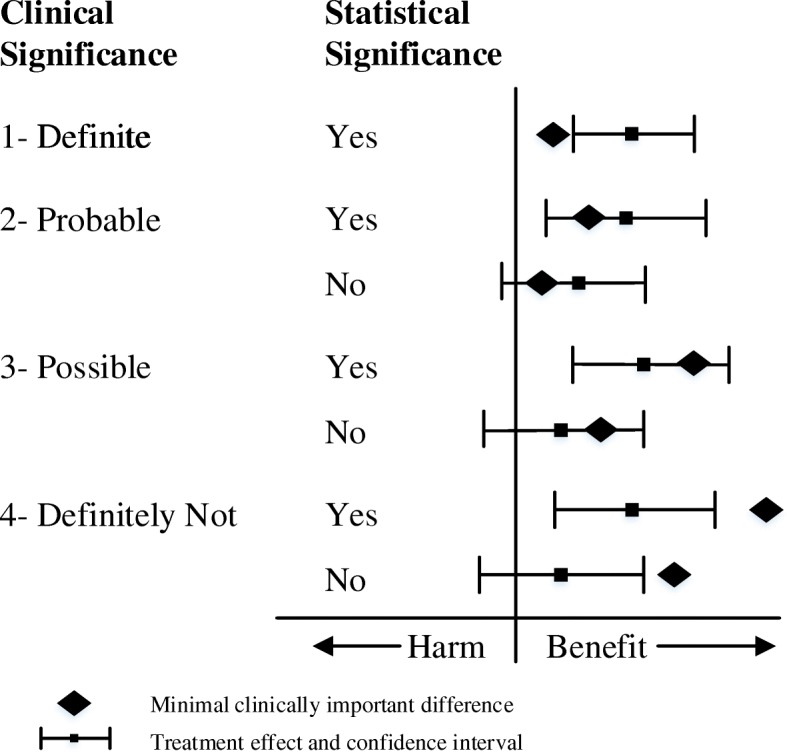
Relationship between clinical significance and statistical significance (adapted from Man-Son-Hing, et al.) [10]

Clinical significance was determined based on the guidelines proposed by Man-Son-Hing et al. [10] which considers the relationship between the MCID of the treatment effect and the CI and designated to one of the following four different levels (Fig. 1): (1) Definite – the MCID is smaller than the lower limit of the CI of the treatment effect, (2) Probable – the MCID is greater than the lower limit of the CI of the treatment effect, but smaller than the treatment effect, (3) Possible – the MCID is less than the upper limit of the CI of the treatment effect, but greater than the treatment effect, and (4) Definitely Not – the MCID is greater than the upper limit of the CI of the treatment effect. The CI should be based on the *α* value specified in the sample size calculation.

We restricted this analysis to randomized questions in superiority trials because these guidelines are intended to be applied to superiority trials. For each study, the delta value was assumed to be the MCID irrespective of whether explicitly stated by the authors. This assumption was applied in an earlier study by Chan et al. [13], and is a pragmatic approach in the context of pediatric oncology. This is based on the premise that the delta value must be reflective of the MCID to the extent that it will result in strong evidence to change standard of care, while also feasible to achieve in a rare disease population. A traditional approach to surveying clinicians and patients to determine a MCID is not realistic in the scope of rare diseases and thus the delta value must follow a definition, which is pragmatic yet clinically relevant and evidence-based. This analysis was restricted to randomized questions to account for studies with multiple primary outcomes. Additionally, only randomized questions were included where the treatment effect and its CI were reported or could be calculated.

### Statistical analysis

The CI of the treatment effect for each randomized question was determined with the methodology outlined by Hackshaw [26] and Altman and Anderson [27] for dichotomous and time-to-event primary outcomes respectively when the CI of the treatment effect was not reported. The CI of the treatment effect for time-to-event outcomes could be calculated only if the CI associated with the experimental and control estimates were reported or a Kaplan-Meier curve with patients at risk was reported. The time point specified in the sample size calculation was used and if it could not be inferred from a Kaplan-Meier curve, or was not reported, the time point reported was used. If the aforementioned was not provided, the CI for the treatment effect could not be calculated and the randomized question was excluded from this analysis. The treatment effect CI was calculated based on the design *α* value if reported in the sample size calculation and if not reported an alpha of 0.05 was assumed. In the event the primary outcome of the sample size calculation included both an absolute and relative difference, the absolute difference was used. The level of concordance between statistical and clinical significance was assessed through descriptive statistics. Descriptive statistics were calculated to assess the reporting of methodological attributes associated with clinical significance. SAS (Statistical Analysis Software) version 9.4 (SAS Institute, Cary, NC, USA) was used to perform all analyses.

## Results

**Fig. 2:**
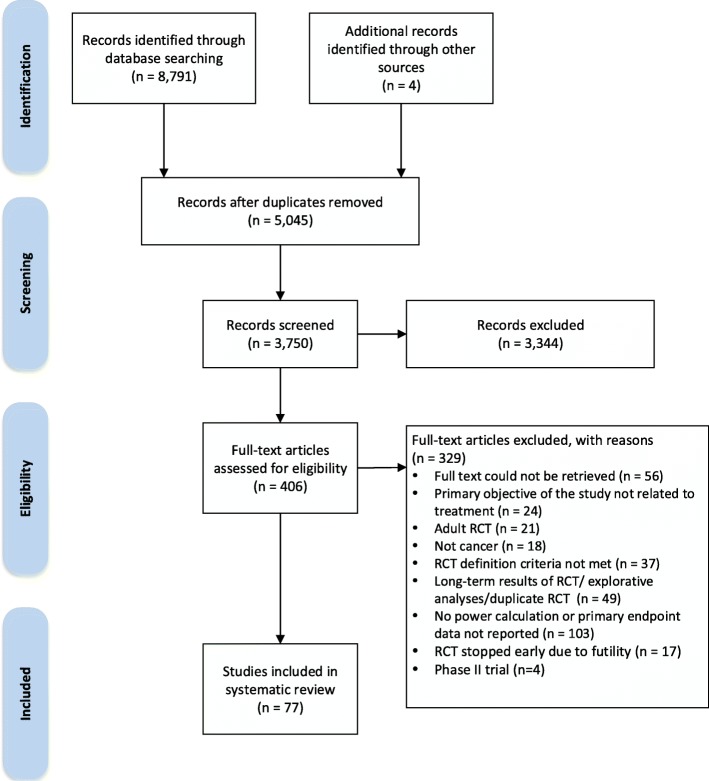
Selection of randomized controlled trials in the systematic review

Our search identified 3750 unique studies from Medline, EMBASE, and the Cochrane Childhood Cancer Group Specialized Register accessed through CENTRAL. Following title and abstract screening, 406 studies were evaluated for eligibility based on full-text review. Of these studies, 329 studies were excluded and 77 studies were included in the systematic review (Fig. 2) (Additional file 1: Appendix B). Table 1 summarizes the characteristics of the included studies.

**Table 1:** Characteristics of 77 included studies, by non-inferiority and superiority trials

Characteristic	Non-inferiority trials (*N* = 11)		Superiority trials (*N* = 66)	
	*n*	%	*n*	%
Journal of publication				
*Journal of Clinical Oncology*	3	27.3	25	37.9
*Blood*	2	18.2	10	15.2
*Pediatric Blood & Cancer*	0	0.0	6	9.1
*Leukemia*	1	9.1	4	6.1
*Lancet*	2	18.2	1	1.5
*Cancer*	0	0.0	4	6.1
*Lancet Oncology*	1	9.1	3	4.5
*New England Journal of Medicine*	1	9.1	2	3
Other	1	9.1	11	16.7
Region of publication				
Europe	6	54.5	23	34.8
North America	4	36.4	41	62.1
Other	1	9.1	2	3
Year of publication				
1976 to 1989	1	9.1	4	6.1
1990 to 2003	4	36.4	28	42.4
2004 to 2016	6	54.5	34	51.5
Source of funding				
Non-industry	10	90.9	56	84.8
Industry and non-industry	0	0.0	2	3
Not stated	1	9.1	8	12.1
Study participants				
Exclusively children	7	63.6	41	62.1
Adults included	4	36.4	25	37.9
Disease site				
Hematological	7	63.6	43	65.2
Central nervous system tumor	1	9.1	11	16.7
Non-central nervous system solid tumor	3	27.3	12	18.2
RCT study design				
2 × 2 factorial	0	0.0	4	6.1
Greater than 2 arms	1	9.1	5	7.6
Two-armed	10	90.9	57	86.3
RCT trial group				
POG	1	9.1	15	22.7
CCG	1	9.1	12	18.2
COG	0	0.0	8	12.1
BFM	1	9.1	9	13.6
UK MRC	1	9.1	4	6.1
Other	7	63.6	18	27.3
Outcome				
Time-to-event	10	90.9	56	84.8
Dichotomous	1	9.1	10	15.2
Intervention in question				
Chemotherapy	9	81.8	57	86.4
Multimodal therapy	0	0.0	2	3
Hemopoietic stem-cell transplant	1	9.1	6	9.1
Radiation therapy	1	9.1	1	1.5

*RCT* randomized control trial, *POG* Pediatric Oncology Group, *CCG* Children’s Cancer Group, *COG* Children’s Oncology Group, *BFM* Berlin Frankfurt Münster Study Group; *UK MRC* United Kingdom Medical Research Council

Table 2 summarizes the methodological attributes relevant to assessing clinical significance for all included studies by randomized questions stratified by RCT type (non-inferiority and superiority RCT, herein, respectively). Only 4.2% (15.4 and 2.4%) of randomized questions explicitly identified that the delta value was based on the MCID, while 22.1% (76.9 and 13.4%) of randomized questions discussed the clinical importance in relation to the delta value specified in their sample size calculation. The majority (95.6% overall; 100.0 and 95.1%) of randomized questions reported the delta value in the sample size calculation as an absolute value and the minority in relative terms (e.g., relative risk reduction, relative hazard rate, etc.). Almost three quarters reported (76.8% overall; 76.9 and 76.8%) the estimate assumed for the control group, of which only 18.9% (46.2 and 14.6%) reported justification for why the estimate was assumed. The statistical significance of the primary outcome was reported using a *p* value in 83.2% (100.0 and 80.5%) of randomized questions, while over half (67.4% overall; 92.3 and 63.4%) reported CIs or standard error bars for the experimental and control values. The majority of studies reported type 1 and type 2 errors in their sample size calculations; however, only 12.6% (46.2 and 7.3%) reported the treatment effect in the results.

**Table 2:** Methodological attributes relevant to interpretation of study results from a clinical perspective for 95 randomized questions, by non-inferiority and superiority trials

Characteristic	Non-inferiority trials (*N* = 13)		Superiority trials (*N* = 82)	
	*n*	%^b^	*n*	%^b^
Methods				
Expected magnitude of difference identified explicitly as the MCID^c^	2	15.4	2	2.4
Justification for MCID^a^				
Clinical relevance	1	50.0	1	50.0
Methodological	1	50.0	1	50.0
Delta value				
Stated as an absolute difference	13	100.0	78	95.1
Margin (median, IQR)	− 0.10	− 0.10,0.10	0.12	0.10, 0.17
Time-to-event	12	92.3	69	88.5
Dichotomous outcome	1	7.7	9	11.5
Stated as relative difference	0	0.0	7	8.5
Margin (median, IQR)	N/A		0.63	0.60, 2.50
Time-to-event	N/A		6	85.7
Dichotomous outcome	N/A		1	14.3
Stated as a percentage and ratio	0	0.0	4	4.9
Anticipated control value stated	10	76.9	63	76.8
Assumptions in the control group	6	46.2	12	14.6
Stated				
Results from previous trial or systematic review	5	83.3	11	91.7
Based on clinical expertise	1	16.7	1	8.3
Type 1 error (*α* value)				
Stated	10	76.9	52	63.4
0.20	0	0.0	1	1.9
0.10	2	20.0	2	3.8
0.05	8	80.0	49	94.2
Sides				
Stated	12	92.3	40	48.8
One-sided	10	83.3	29	72.5
Two-sided	2	16.7	11	27.5
Type 2 error (1 − *β* value)				
Stated	12	92.3	81	98.8
< 80%	2	16.7	7	8.6
80 to 85%	6	50.0	58	71.6
85 to 90%	1	8.3	7	8.6
≥ 90%	3	25.0	9	11.1
Results				
Statistical significance of primary outcome reported via *p* value	13	100.0	66	80.5
Confidence intervals/standard error for primary outcome reported	12	92.3	52	63.4
Treatment effect stated	6	46.2	6	7.3
Discussion (and/or Results)				
Clinical importance of primary outcome discussed	10	76.9	11	13.4

^a^*MCID* minimally clinically important difference, *IQR* interquartile range

^b^Percentages may not sum to 100% due to rounding

^c^Assumed to be the delta value from the sample size calculation

**Fig. 3:**
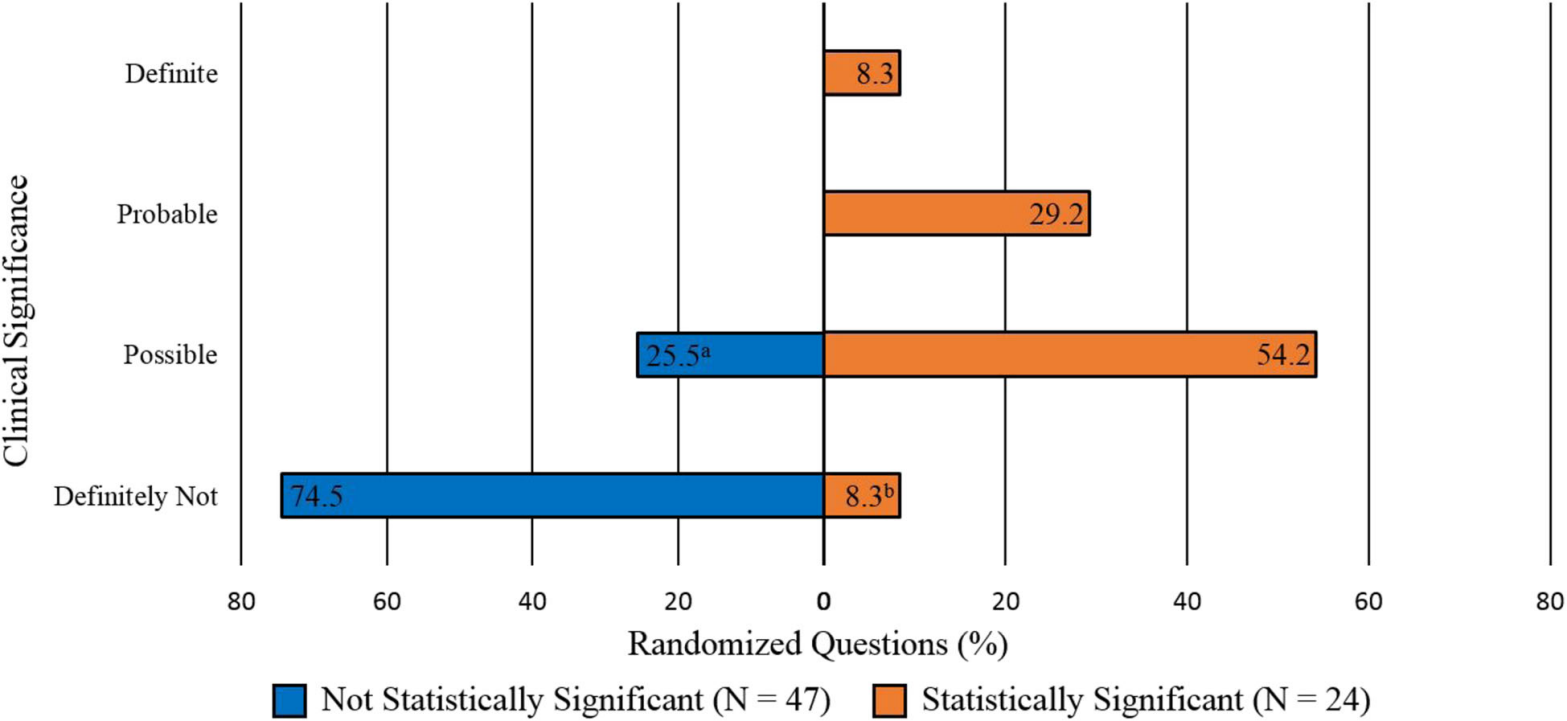
Relationship between statistical significance and clinical significance in superiority randomized controlled trials (RCTs). ^a^Two randomized questions included where the confidence interval of the treatment effect was based on an alpha of 0.05 although the sample size calculation stated an alpha of 0.10. This was due to insufficient information being reported which precluded calculating the 90% confidence interval. ^b^Statistically significant solely due to the direction of the direction of the effect being related to harm as opposed to benefit

Table 3 and Fig. 3 summarize the level of clinical significance in superiority trials, determined when examining the relationship between the MCID of the treatment effect and its associated CIs, in relation to the reporting of statistically significant findings for superiority RCTs that satisfied the criteria. Of the 71 randomized questions that reported statistically insignificant findings, 25.5% (*n* = 12) were found to have possible clinical significance. Of the 24 randomized questions that reported statistically significant findings, 8.3% (*n* = 2) were found to have clinical significance categorized as “Definitely Not,” 83.4% (*n* = 20) as “Probable or Possible,” and 8.3% (*n* = 2) as “Definite.” Of the total 71 randomized questions, only 2.8% (*n* = 2) were found to have definite clinical importance while 45.1% (*n* = 32) were found to have “Probable or Possible” clinical significance and the remaining 52.1% (*n* = 37) were “Definitely Not” clinically significant.

**Table 3:** Relationship between statistical significance and clinical significance in superiority randomized controlled trials consisting of 71 randomized questions

Clinical significance	Statistical significance				Total *N* = 71	
	No (*n* = 47)		Yes (*n* = 24)			
	*N*	%	*N*	%	*N*	%
Definite	0	0.0	2	8.3	2	2.8
Probable	0	0.0	7	29.2	7	9.9
Possible	12^a^	25.5	13	54.2	25	35.2
Definitely Not	35	74.5	2^b^	8.3	37	52.1

^a^Two randomized question included where the confidence interval of the treatment effect was based on an alpha of 0.05 although the sample size calculation stated an alpha of 0.10. This was due to insufficient information being reported which precluding calculating the 90% confidence interval

^b^Statistically significant solely due to the direction of the effect being related to harm as opposed to benefit

## Discussion

In this systematic review, we demonstrated that only a minority of the 77 RCTs (11 non-inferiority and 66 superiority RCTs) in the published pediatric oncology treatment literature reported methodological attributes related to clinical significance. A notable portion of RCTs reporting statistically insignificant results was found to have possible clinical significance and likewise for those reporting statistically significant results.

### Strengths and weaknesses

The strengths of this study stem from the inclusion of all pediatric oncology RCTs, from database inception to July 2016, that evaluated a range of cancer treatments for various cancer types. To our knowledge, this is the first study to assess clinical significance in the rare disease context of pediatric cancer RCTs. A limitation is that the search was restricted to studies in English and was inclusive of the published literature, and was thus, prone to language and publication bias. The assessment of clinical significance was based on the delta value in the published report and not the trial protocol and, therefore, it is possible that information recorded as not reported was reported in the trial protocol. However, the parameters used in this review comprise of CONSORT-mandated items and thus should be reported in the published report. A limitation associated with assessing clinical significance as per Man-Son-Hing et al., [10] is that the weight for each level of clinical significance will vary depending on the research question. However, the definitions applied to classify the level of clinical significance are arbitrary and not meant to be adhered to strictly. For example, in the context of a disease with no available effective treatments, a treatment found to be statistically significant but that only shows possible clinical significance would likely warrant implementation in the clinical setting. This is of particular relevance to pediatric cancer, where some cancer subtypes still have dismal survival and high relapse rates. Conversely, demonstrating superiority to a well-established treatment would require adherence to a strict definition of clinical importance because changes to recommended treatment should not proceed unless definite clinical significance is demonstrated. In interpreting the study findings, it is also important to note that only 13 of the 95 randomized questions were from non-inferiority trials, representing a small minority. Thus, it is critical that the assessments of these non-inferiority trials in this research not be over-interpreted or generalized to the superiority trials.

### Comparison with existing literature

Based on our analysis, the majority (77.9%) of randomized questions did not describe the clinical importance of their findings in relation to the delta value of the interventions in question. The minority (4.2%) of randomized questions explicitly identified the delta value as the MCID with justification. These findings are in line with the limited number of previous studies investigating clinical significance reporting, wherein under-reporting was found [13, 19, 20, 28, 29]. Chan et al. [13] found that, among a random sample of 27 RCTs in major medical journals, 20 articles included sample size calculations, 90% of which reported a delta value but only 11% stated that the delta value was chosen to reflect the MCID of the intervention. Study results were interpreted from the perspective of clinical importance in 20 of 27 (74%) articles, with only one article discussing clinical importance in relation to a reported sample size MCID. In a review of 57 dementia drug RCTs, Molnar et al. [29] found that 46% discussed the clinical significance of their results, and no studies used formally derived MCIDs. These results are in line with our review findings.

### Study explanations and implications

In our study, the failure to incorporate a MCID into study design and/or state whether or not the delta value in the sample size calculation was based on the MCID could, in part, be attributed to poor reporting in combination with the difficulty of achieving a reasonable sample size, even when recruiting patients from multiple institutions and over a long duration of time [30–32]. The formidable challenges of conducting pediatric oncology trials include parent and physician reluctance to involve children in trials, difficulties obtaining consent, permission and assent for study participation, and the dedicated time and attention required to educate children and families, not to mention the remarkably complex logistics of involving multiple sites in multiple countries and the stringent safety monitoring required [33, 34]. Therefore, sample size calculations are perhaps often based on study feasibility and a larger delta value is chosen to reduce the sample size required for the study. This has the potential to place patients at risk of participating in a trial that might lead to erroneous conclusions based on flawed study design and risks the mismanagement of precious time and resources [35]. Often times, studies report that the intervention groups do not differ, when in actuality they lacked sufficient power to support this claim as well as detect a clinically meaningful treatment effect [11, 36–39]. This is concerning in our review wherein about two thirds of the randomized questions in superiority trials were not statistically significant, yet 25.5% were found to be have probable or possible clinical significance. Arguably, it is unreasonable to expect for RCTs to be purely powered based on the MCID with disregard for feasibility issues; however, trials would be improved if they were powered with consideration of the MCID as well as feasibility, as recommended in the CONSORT Statement [11]. This point is relevant in rare disease trials where a MCID purely reflective of clinician and patient preferences is not realistic. Rather, the MCID in a rare disease context is perhaps best determined by weighing the evidence in the literature and/or pilot studies, clinical expertise, and patient preferences in combination with feasibility. Careful evaluation of the aforementioned would help ensure that evidence-based decisions to change clinical practice are supported.

As Cook et al. [24] state, improved standards in both RCT sample size calculations and reporting of these calculations could assist health care professionals, patients, researchers and funders to judge the strength of the available evidence and ensure responsible use of scarce resources. Without explicit discussion of the treatment effect in relation to the MCID, we allow for subjective interpretation of trial results based solely on whether or not the results were statistically significant. Statistical significance determined by a *p* value only provides information on whether a significant difference exists and does not provide information on the direction and size of the effect [14]. For instance, if a decision-maker relies solely on statistical significance, they are only able to infer that an experimental treatment is significantly different, or not, from the control, based on the power of the study. However, if a decision-maker relies on clinical significance, which also provides information on statistical significance, they can infer the direction and size of this difference in relation to a MCID (by comparing where the treatment effect and its CI fall in relation to the MCID). The latter approach provides greater utility because a decision-maker can ascertain how harmful or beneficial an experimental treatment is in comparison to the control and assess the value of a trial with greater confidence, whether deemed statistically significant or not.

A study might be statistically significant based on an arbitrary delta value and conclusions might be drawn without any consideration for the precision of the treatment effect and whether it was clinically meaningful. This was demonstrated in our study where studies found to be statistically significant but definitely not clinically significant were due to the fact that the direction of the effect size was related to harm as opposed to benefit, which cannot be ascertained solely through a *p* value. Additionally, a notable portion of studies found to be statistically insignificant were found to have possible clinical significance, which demonstrates, as stated in the CONSORT Statement, that statistically insignificant results do not preclude potential clinically meaningful findings.

In addition to designing a study based on a delta value reflective of a MCID, it is necessary to provide justification of the MCID, experimental value and control value, which our study revealed were only reported by a minority of studies. Reporting of this justification will allow the reader to apply the appropriate weight to the authors’ conclusions as values based on systematic reviews or meta-analyses will have a higher weight than values based on the research team’s expertise. It is also essential for the treatment effect to be interpreted with the precision of the CI in mind [10, 14]. For instance, a treatment effect may be within the MCID, but due to inadequate power from an inaccurate sample size calculation, the precision of this estimate may be weak and thus the findings should be graded as low evidence. CIs should be reported for the experimental and control values as well as the treatment effect as recommended by the CONSORT Statement [11].

### Recommendations

Given our study results and the implications discussed, in Table 4 we propose recommendations, adapted and informed by Cook et al. [24], Moher et al. [11], and Koynova et al. [25] to promote the incorporation of clinical significance into RCT design and interpretation. Our results also raise the question of whether clinical significance is under-utilized and poorly reported in other rare disease contexts wherein obtaining an adequate study sample challenges feasibility. Moreover, research and knowledge translation efforts are required to raise awareness and understanding of the importance of clinical significance.

**Table 4:** Recommendations for incorporating clinical significance into randomized controlled trial design and interpretation

Recommendations^a^	
1. Conduct a comprehensive review of the literature to identify the MCID. If the RCT is completely novel, use preliminary pilot data to inform the MCID	
2. Perform a sample size calculation using a delta value that is based on the MCID. If the sample size is not feasible given resource constraints, adjust the delta value to increase the sample size to a value that is still clinically meaningful	
3. When reporting the results of an RCT ensure the following are reported in the sample size calculation: • Type 1 error (*α* value) ○ One- or two-sided *p* value • Type 2 error (*β* value) ○ At least 80% is recommended • Estimated controlled value and justification • Estimated experimental value and justification • Delta value in absolute terms and justification of treatment effect • Explicitly identify primary outcome when multiple outcomes are being investigated	
4. Calculate and report confidence intervals for the experimental and controlled values as well as the treatment effect	
5. Interpret the treatment effect and its confidence interval in relation to the MCID and place weight on conclusions based on the precision determined by the confidence interval	
6. Ensure conclusions reflect the quality of the trial based on the recommendations of the CONSORT Statement	

*MCID* minimally clinically important difference, *RCT* randomized control trial, *CONSORT* Consolidated Standards of Reporting Trials

^a^Recommendations adapted from and informed by Cook et al. [24], Moher et al. [11], and Koynova et al. [25]

## Additional file

Additional file 1: Comprehensive search strategy data. (DOCX 66 kb)

## Abbreviations

CI: Confidence interval
CONSORT: Consolidated Standards of Reporting Trials
MCID: Minimal clinically important difference
PRISMA: Preferred Reporting Items for Systematic Reviews and Meta-Analyses
RCT: Randomized controlled trial

## References

[1] Canadian Cancer Society’s Advisory Committee on Cancer Statistics (2015). Canadian Cancer Statistics 2015.

[2] American Cancer Society (2017). Cancer Facts & Figures 2017.

[3] O’Leary M, Krailo M, Anderson JR, et al. Progress in childhood cancer: 50 years of research collaboration, a report from the Children’s Oncology Group. Semin Oncol 2008: Abstract 35, p. 484–493. Elsevier. 10.1053/j.seminoncol.2008.07.008 PMC2702720 18929147

[4] Bleyer A, Budd T, Montello M (2006). Adolescents and young adults with cancer: the scope of the problem and criticality of clinical trials. Cancer.

[5] Joseph P. D., Craig J. C., Tong A., Caldwell P. H. Y. (2016). Researchers, Regulators, and Sponsors Views on Pediatric Clinical Trials: A Multinational Study. PEDIATRICS.

[6] Pritchard-Jones K, Lewison G, Camporesi S (2011). The state of research into children with cancer across Europe: new policies for a new decade. Ecancermedicalscience.

[7] Akobeng AK (2008). Confidence intervals and p-values in clinical decision making. Acta Paediatr.

[8] Vora A, Goulden N, Wade R (2013). Treatment reduction for children and young adults with low-risk acute lymphoblastic leukaemia defined by minimal residual disease (UKALL 2003): a randomised controlled trial. Lancet Oncol.

[9] Yu AL, Gilman AL, Ozkaynak MF (2010). Anti-GD2 antibody with GM-CSF, interleukin-2, and isotretinoin for neuroblastoma. N Engl J Med.

[10] Man-Son-Hing M, Laupacis A, O’Rourke K (2002). Determination of the clinical importance of study results. J Gen Intern Med.

[11] Moher D, Hopewell S, Schulz KF (2012). CONSORT 2010 explanation and elaboration: updated guidelines for reporting parallel group randomised trials. Int J Surg.

[12] Jaeschke R, Singer J, Guyatt GH (1989). Measurement of health status. Ascertaining the minimal clinically important difference. Control Clin Trials.

[13] Chan KB, Man-Son-Hing M, Molnar FJ (2001). How well is the clinical importance of study results reported? An assessment of randomized controlled trials. CMAJ.

[14] Ferrill MJ, Brown DA, Kyle JA (2010). Clinical versus statistical significance: interpreting P values and confidence intervals related to measures of association to guide decision making. J Pharm Pract.

[15] David MC (2006). How to make clinical decisions from statistics. Clin Exp Optom.

[16] Pocock SJ, Hughes MD, Lee RJ (1987). Statistical problems in the reporting of clinical trials. A survey of three medical journals. N Engl J Med.

[17] Gardner MJ, Altman DG (1986). Confidence intervals rather than P values: estimation rather than hypothesis testing. Br Med J (Clin Res Ed).

[18] Bland JM, Peacock JL (2002). Interpreting statistics with confidence. The Obstetrician & Gynaecologist.

[19] Hoffmann TC, Thomas ST, Shin PN (2014). Cross-sectional analysis of the reporting of continuous outcome measures and clinical significance of results in randomized trials of non-pharmacological interventions. Trials.

[20] van Tulder M, Malmivaara A, Hayden J (2007). Statistical significance versus clinical importance: trials on exercise therapy for chronic low back pain as example. Spine.

[21] Moher D, Liberati A, Tetzlaff J (2009). Preferred reporting items for systematic reviews and meta-analyses: the PRISMA statement. PLoS Med.

[22] CADTH. Strings attached: CADTH database search filters [Internet]. https://www.cadth.ca/resources/finding-evidence. Accessed 26 Sept 2018.

[23] Leclercq E, Leeflang MM, van Dalen EC (2013). Validation of search filters for identifying pediatric studies in PubMed. J Pediatr.

[24] Cook JA, Hislop J, Altman DG (2015). Specifying the target difference in the primary outcome for a randomised controlled trial: guidance for researchers. Trials.

[25] Koynova D, Lühmann R, Fischer R (2013). A framework for managing the minimal clinically important difference in clinical trials. Ther Innov Regul Sci.

[26] Hackshaw A (2009). Statistical formulae for calculating some 95% confidence intervals. A concise guide to clinical trials.

[27] Altman DG, Andersen PK (1999). Calculating the number needed to treat for trials where the outcome is time to an event. BMJ.

[28] Castellini G, Gianola S, Bonovas S (2016). Improving power and sample size calculation in rehabilitation trial reports: a methodological assessment. Arch Phys Med Rehabil.

[29] Molnar FJ, Man-Son-Hing M, Fergusson D (2009). Systematic review of measures of clinical significance employed in randomized controlled trials of drugs for dementia. J Am Geriatr Soc.

[30] PHY C, Murphy SB, Butow PN, et al. Clinical trials in children. Lancet. 364(9436):803–11. 10.1016/S0140-6736(04)16942-0 15337409

[31] Estlin EJ, Ablett S (2001). Practicalities and ethics of running clinical trials in paediatric oncology - the UK experience. Eur J Cancer.

[32] Burke ME, Albritton K, Marina N (2007). Challenges in the recruitment of adolescents and young adults to cancer clinical trials. Cancer.

[33] Joseph PD, Craig JC, Caldwell PH (2015). Clinical trials in children. Br J Clin Pharmacol.

[34] Berg SL (2007). Ethical challenges in cancer research in children. Oncologist.

[35] Detsky AS (1985). Using economic analysis to determine the resource consequences of choices made in planning clinical trials. J Chronic Dis.

[36] Moher D, Dulberg CS, Wells GA (1994). Statistical power, sample size, and their reporting in randomized controlled trials. JAMA.

[37] Freiman JA, Chalmers TC, Smith H (1978). The importance of beta, the type II error and sample size in the design and interpretation of the randomized control trial. Survey of 71 “negative” trials. N Engl J Med.

[38] Charles P., Giraudeau B., Dechartres A., Baron G., Ravaud P. (2009). Reporting of sample size calculation in randomised controlled trials: review. BMJ.

[39] Yusuf S, Collins R, Peto R (1984). Why do we need some large, simple randomized trials?. Stat Med.

