# Supplementary material for: Clinical significance in pediatric oncology randomized controlled treatment trials: a systematic review
Source: Trials. 2018 Oct 5;19:539. doi: 10.1186/s13063-018-2925-8 (PMC6173909; doi:10.1186/s13063-018-2925-8)
Supplement: Supplementary file 1 — Comprehensive search strategy data. (DOCX 66 kb) [file 13063_2018_2925_MOESM1_ESM.docx]

***Appendix A***

**Search Strategies**

*EMBASE*

1. Randomized Controlled Trial.pt. or Pragmatic Clinical Trial.pt. or exp Randomized Controlled Trials as Topic/ or Randomized Controlled Trial (Topic) or Randomized Controlled Trial/ or Randomization/ or Random Allocation/ or Double-Blind Method/ or Double-Blind Procedure or Double-Blind Studies/ or Single-Blind Method/ or Single-Blind Procedure/ or Single-Blind Studies/ or Placebos/ or Placebo/ or (random* or sham or placebo*).ti,ab,hw,kf,kw. or ((singl* or doubl*) adj (blind* or dumm* or mask*)).ti,ab,hw,kf,kw. rr ((tripl* or trebl*) adj (blind* or dumm* or mask*)).ti,ab,hw,kf,kw.
2. leukemia or leukemi* or leukaemi* or (childhood ALL) or AML or lymphoma or lymphom* or

hodgkin OR hodgkin* or T-cell or B-cell or non-hodgkin or sarcoma or sarcom* or sarcoma, Ewing's or Ewing* or osteosarcoma or osteosarcom* or wilms tumor or wilms* or nephroblastom* or neuroblastoma or neuroblastom* or rhabdomyosarcoma or rhabdomyosarcom* or teratoma or teratom* or hepatoma or hepatom* or hepatoblastoma or hepatoblastom* or PNET or medulloblastoma or medulloblastom* or PNET* or neuroectodermal tumors, primitive or retinoblastoma or retinoblastom* or meningioma or meningiom* or glioma or gliom* or pediatric oncology or paediatric oncology or childhood cancer or childhood tumor or childhood tumors or brain tumor* or brain tumour* or brain neoplasms or central nervous system neoplasm or central nervous system neoplasms or central nervous system tumor* or central nervous system tumour* or brain cancer* or brain neoplasm* or intracranial neoplasm* or leukemia lymphocytic acute or acute lymphoblastic leukemia/

1. cancer or cancers or cancer* or oncology or oncolog* or neoplasm or neoplasms or neoplasm* or

carcinoma or carcinom* or tumor or tumour or tumor* or tumour* or tumors or tumours or

malignan* or malignant or hematooncological or hemato oncological or hemato-oncological or

hematologic neoplasms or hematolo*

1. 1 AND 2 AND 3
2. Limit 4 to Human/ English Language
3. Limit 5 to ("all infant (birth to 23 months)" or "all child (0 to 18 years)" or "newborn infant (birth to 1 month)" or "infant (1 to 23 months)" or "preschool child (2 to 5 years)" or "child (6 to 12 years)" or "adolescent (13 to 18 years)")
4. Final filter: Limit 7 to NOT IN MEDLINE

*MEDLINE*

1. Randomized Controlled Trial.pt. or Pragmatic Clinical Trial.pt. or exp Randomized Controlled Trials as Topic/ or Randomized Controlled Trial (Topic) or Randomized Controlled Trial/ or Randomization/ or Random Allocation/ or Double-Blind Method/ or Double-Blind Procedure or Double-Blind Studies/ or Single-Blind Method/ or Single-Blind Procedure/ or Single-Blind Studies/ or Placebos/ or Placebo/ or (random* or sham or placebo*).ti,ab,hw,kf,kw. or ((singl* or doubl*) adj (blind* or dumm* or mask*)).ti,ab,hw,kf,kw. rr ((tripl* or trebl*) adj (blind* or dumm* or mask*)).ti,ab,hw,kf,kw.
2. leukemia or leukemi* or leukaemi* or (childhood ALL) or AML or lymphoma or lymphom* or

hodgkin OR hodgkin* or T-cell or B-cell or non-hodgkin or sarcoma or sarcom* or sarcoma, Ewing's or Ewing* or osteosarcoma or osteosarcom* or wilms tumor or wilms* or nephroblastom* or neuroblastoma or neuroblastom* or rhabdomyosarcoma or rhabdomyosarcom* or teratoma or teratom* or hepatoma or hepatom* or hepatoblastoma or hepatoblastom* or PNET or medulloblastoma or medulloblastom* or PNET* or neuroectodermal tumors, primitive or retinoblastoma or retinoblastom* or meningioma or meningiom* or glioma or gliom* or pediatric oncology or paediatric oncology or childhood cancer or childhood tumor or childhood tumors or brain tumor* or brain tumour* or brain neoplasms or central nervous system neoplasm or central nervous system neoplasms or central nervous system tumor* or central nervous system tumour* or brain cancer* or brain neoplasm* or intracranial neoplasm* or leukemia lymphocytic acute or acute lymphoblastic leukemia/

1. cancer or cancers or cancer* or oncology or oncolog* or neoplasm or neoplasms or neoplasm* or

carcinoma or carcinom* or tumor or tumour or tumor* or tumour* or tumors or tumours or

malignan* or malignant or hematooncological or hemato oncological or hemato-oncological or

hematologic neoplasms or hematolo*

1. 1 AND 2 AND 3
2. Limit 4 to Human/ English Language
3. Limit 5 to ("all infant (birth to 23 months)" or "all child (0 to 18 years)" or "newborn infant (birth to 1 month)" or "infant (1 to 23 months)" or "preschool child (2 to 5 years)" or "child (6 to 12 years)" or "adolescent (13 to 18 years)")

*CENTRAL (Wiley)*

1. SR-CHILDCA

***Appendix B***

**List of Included Studies:**

1. Evans AE, Albo V, D'Angio GJ. Cyclophosphamide treatment of patients with localized and regional neuroblastoma. A randomized study. Cancer 1976:38(2):655-659.

2. Hvizdala EV, Berard C, Callihan T, et al. Lymphoblastic lymphoma in children--a randomized trial comparing LSA2-L2 with the A-COP+ therapeutic regimen: a Pediatric Oncology Group Study. Journal of Clinical Oncology 1988:6(1):26-33.

3. Maurer HM, Beltangady M, Gehan EA, et al. The Intergroup Rhabdomyosarcoma Study-I. A final report. Cancer 1988:61(2):209-220.

4. Meadows AT, Sposto R, Jenkin RD, et al. Similar efficacy of 6 and 18 months of therapy with four drugs (COMP) for localized non-Hodgkin's lymphoma of children: a report from the Childrens Cancer Study Group. Journal of Clinical Oncology 1989:7(1):92-99.

5. Van Eys J, Berry D, Crist W, et al. Treatment intensity and outcome for children with acute lymphocytic leukemia of standard risk. A Pediatric Oncology Group Study. Cancer 1989:63(8):1466-1471.

6. Patte C, Philip T, Rodary C, et al. High survival rate in advanced-stage B-cell lymphomas and leukemias without CNS involvement with a short intensive polychemotherapy: results from the French Pediatric Oncology Society of a randomized trial of 216 children. Journal of Clinical Oncology 1991:9(1):123-132.

7. Tournade MF, Com-Nougue C, Voute PA, et al. Results of the Sixth International Society of Pediatric Oncology Wilms' Tumor Trial and Study: a risk-adapted therapeutic approach in Wilms' tumor. Journal of Clinical Oncology 1993:11(6):1014-1023.

8. Tubergen DG, Gilchrist GS, O'Brien RT, et al. Improved outcome with delayed intensification for children with acute lymphoblastic leukemia and intermediate presenting features: a Childrens Cancer Group phase III trial. Journal of Clinical Oncology 1993:11(3):527-537.

9. Finlay JL, Boyett JM, Yates AJ, et al. Randomized phase III trial in childhood high-grade astrocytoma comparing vincristine, lomustine, and prednisone with the eight-drugs-in-1-day regimen. Childrens Cancer Group. Journal of Clinical Oncology 1995:13(1):112-123.

10. Lange BJ, Blatt J, Sather HN, et al. Randomized comparison of moderate-dose methotrexate infusions to oral methotrexate in children with intermediate risk acute lymphoblastic leukemia: a Childrens Cancer Group study. Medical & Pediatric Oncology 1996:27(1):15-20.

11. Ravindranath Y, Yeager AM, Chang MN, et al. Autologous bone marrow transplantation versus intensive consolidation chemotherapy for acute myeloid leukemia in childhood. Pediatric Oncology Group. New England Journal of Medicine 1996:334(22):1428-1434.

12. Woods WG, Kobrinsky N, Buckley JD, et al. Timed-sequential induction therapy improves postremission outcome in acute myeloid leukemia: a report from the Children's Cancer Group. Blood 1996:87(12):4979-4989.

13. Link MP, Shuster JJ, Donaldson SS, et al. Treatment of children and young adults with early-stage non-hodgkin's lymphoma. New England Journal of Medicine 1997:337(18):1259-1266.

14. Green DM, Breslow NE, Beckwith JB, et al. Comparison between single-dose and divided-dose administration of dactinomycin and doxorubicin for patients with Wilms' tumor: a report from the National Wilms' Tumor Study Group. Journal of Clinical Oncology 1998:16(1):237-245.

15. Harris MB, Shuster JJ, Pullen DJ, et al. Consolidation therapy with antimetabolite-based therapy in standard-risk acute lymphocytic leukemia of childhood: a Pediatric Oncology Group Study. Journal of Clinical Oncology 1998:16(8):2840-2847.

16. Mahoney DH, Jr., Shuster J, Nitschke R, et al. Intermediate-dose intravenous methotrexate with intravenous mercaptopurine is superior to repetitive low-dose oral methotrexate with intravenous mercaptopurine for children with lower-risk B-lineage acute lymphoblastic leukemia: a Pediatric Oncology Group phase III trial. Journal of Clinical Oncology 1998:16(1):246-254.

17. Pratt CB, Maurer HM, Gieser P, et al. Treatment of unresectable or metastatic pediatric soft tissue sarcomas with surgery, irradiation, and chemotherapy: a Pediatric Oncology Group study. Medical & Pediatric Oncology 1998:30(4):201-209.

18. Pratt CB, Pappo AS, Gieser P, et al. Role of adjuvant chemotherapy in the treatment of surgically resected pediatric nonrhabdomyosarcomatous soft tissue sarcomas: A Pediatric Oncology Group Study. Journal of Clinical Oncology 1999:17(4):1219-1226.

19. Zeltzer PM, Boyett JM, Finlay JL, et al. Metastasis stage, adjuvant treatment, and residual tumor are prognostic factors for medulloblastoma in children: conclusions from the Children's Cancer Group 921 randomized phase III study. Journal of Clinical Oncology 1999:17(3):832-845.

20. Buchanan GR, Rivera GK, Pollock BH, et al. Alternating drug pairs with or without periodic reinduction in children with acute lymphoblastic leukemia in second bone marrow remission: a Pediatric Oncology Group Study. Cancer 2000:88(5):1166-1174.

21. Hann I, Vora A, Richards S, et al. Benefit of intensified treatment for all children with acute lymphoblastic leukaemia: results from MRC UKALL XI and MRC ALL97 randomised trials. UK Medical Research Council's Working Party on Childhood Leukaemia. Leukemia 2000:14(3):356-363.

22. Harris MB, Shuster JJ, Pullen J, et al. Treatment of children with early pre-B and pre-B acute lymphocytic leukemia with antimetabolite-based intensification regimens: A pediatric oncology group study. Leukemia 2000:14(9):1570-1576.

23. Locatelli F, Zecca M, Rondelli R, et al. Graft versus host disease prophylaxis with low-dose cyclosporine-A reduces the risk of relapse in children with acute leukemia given HLA- identical sibling bone marrow transplantation: Results of a randomized trial. Blood 2000:95(5):1572-1579.

24. Mahoney DH, Jr., Shuster JJ, Nitschke R, et al. Intensification with intermediate-dose intravenous methotrexate is effective therapy for children with lower-risk B-precursor acute lymphoblastic leukemia: A Pediatric Oncology Group study. Journal of Clinical Oncology 2000:18(6):1285-1294.

25. Ortega JA, Douglass EC, Feusner JH, et al. Randomized comparison of cisplatin/vincristine/fluorouracil and cisplatin/continuous infusion doxorubicin for treatment of pediatric hepatoblastoma: A report from the Children's Cancer Group and the Pediatric Oncology Group. Journal of Clinical Oncology 2000:18(14):2665-2675.

26. Schrappe M, Reiter A, Ludwig WD, et al. Improved outcome in childhood acute lymphoblastic leukemia despite reduced use of anthracyclines and cranial radiotherapy: results of trial ALL-BFM 90. German-Austrian-Swiss ALL-BFM Study Group. Blood 2000:95(11):3310-3322.

27. Creutzig U, Ritter J, Zimmermann M, et al. Idarubicin improves blast cell clearance during induction therapy in children with AML: Results of study AML-BFM 93. Leukemia 2001:15(3):348-354.

28. Creutzig U, Ritter J, Zimmermann M, et al. Improved treatment results in high-risk pediatric acute myeloid leukemia patients after intensification with high-dose cytarabine and mitoxantrone: Results of study acute myeloid Leukemia-Berlin-Frankfurt-Munster 93. Journal of Clinical Oncology 2001:19(10):2705-2713.

29. Donaldson SS, Meza J, Breneman JC, et al. Results from the IRS-IV randomized trial of hyperfractionated radiotherapy in children with rhabdomyosarcoma--a report from the IRSG. International journal of radiation oncology, biology, physics 2001:51(3):718-728.

30. Rizzari C, Valsecchi MG, Arico M, et al. Effect of protracted high-dose L-asparaginase given as a second exposure in a Berlin-Frankfurt-Munster-based treatment: Results of the randomized 9102 intermediate-risk childhood acute lymphoblastic leukemia study - A report from the Associazione Italiana Ematologia Oncologia Pediatrica. Journal of Clinical Oncology 2001:19(5):1297-1303.

31. Sposto R, Meadows AT, Chilcote RR, et al. Comparison of long-term outcome of children and adolescents with disseminated non-lymphoblastic non-hodgkin lymphoma treated with COMP or daunomycin-comp: A report from the children's cancer group. Medical and pediatric oncology 2001:37(5):432-441.

32. Tournade MF, ComNougue C, De Kraker J, et al. Optimal duration of preoperative therapy in unilateral and nonmetastatic Wilms' tumor in children older than 6 months: Results of the Ninth International Society of Pediatric Oncology Wilms' Tumor Trial and Study. Journal of Clinical Oncology 2001:19(2):488-500.

33. Duval M, Suciu S, Ferster A, et al. Comparison of Escherichia coli-asparaginase with Erwinia-asparaginase in the treatment of childhood lymphoid malignancies: results of a randomized European Organisation for Research and Treatment of Cancer-Children's Leukemia Group phase 3 trial. Blood 2002:99(8):2734-2739.

34. Lange BJ, Bostrom BC, Cherlow JM, et al. Double-delayed intensification improves event-free survival for children with intermediate-risk acute lymphoblastic leukemia: a report from the Children's Cancer Group. Blood 2002:99(3):825-833.

35. Laver JH, Mahmoud H, Pick TE, et al. Results of a randomized phase III trial in children and adolescents with advanced stage diffuse large cell non-Hodgkin's lymphoma: a Pediatric Oncology Group study. Leukemia & lymphoma 2002:43(1):105-109.

36. Bostrom BC, Sensel MR, Sather HN, et al. Dexamethasone versus prednisone and daily oral versus weekly intravenous mercaptopurine for patients with standard-risk acute lymphoblastic leukemia: a report from the Children's Cancer Group. Blood 2003:101(10):3809-3817.

37. de Kraker J, Graf N, van Tinteren H, et al. Reduction of postoperative chemotherapy in children with stage I intermediate-risk and anaplastic Wilms' tumour (SIOP 93-01 trial): a randomised controlled trial. Lancet 2004:364(9441):1229-1235.

38. Hill FGH, Richards S, Gibson B, et al. Successful treatment without cranial radiotherapy of children receiving intensified chemotherapy for acute lymphoblastic leukaemia: results of the risk-stratified randomized central nervous system treatment trial MRC UKALL XI (ISRC TN 16757172). British journal of haematology 2004:124(1):33-46.

39. Taylor RE, Bailey CC, Robinson KJ, et al. Impact of radiotherapy parameters on outcome in the International Society of Paediatric Oncology/United Kingdom Children's Cancer Study Group PNET-3 study of preradiotherapy chemotherapy for M0-M1 medulloblastoma. International journal of radiation oncology, biology, physics 2004:58(4):1184-1193.

40. Balduzzi A, Valsecchi MG, Uderzo C, et al. Chemotherapy versus allogeneic transplantation for very-high-risk childhood acute lymphoblastic leukaemia in first complete remission: Comparison by genetic randomisation in an international prospective study. Lancet 2005:366(9486):635-642.

41. Geyer JR, Sposto R, Jennings M, et al. Multiagent chemotherapy and deferred radiotherapy in infants with malignant brain tumors: a report from the Children's Cancer Group. Journal of Clinical Oncology 2005:23(30):7621-7631.

42. Mitchell CD, Richards SM, Kinsey SE, et al. Benefit of dexamethasone compared with prednisolone for childhood acute lymphoblastic leukaemia: Results of the UK Medical Research Council ALL97 randomized trial. British journal of haematology 2005:129(6):734-745.

43. Pritchard J, Cotterill SJ, Germond SM, et al. High dose melphalan in the treatment of advanced neuroblastoma: Results of a randomised trial (ENSG-1) by the European Neuroblastoma Study Group. Pediatric Blood and Cancer 2005:44(4):348-357.

44. van der Werff ten Bosch J, Suciu S, Thyss A, et al. Value of intravenous 6-mercaptopurine during continuation treatment in childhood acute lymphoblastic leukemia and non-Hodgkin's lymphoma: Final results of a randomized phase III trial (58881) of the EORTC CLG. Leukemia 2005:19(5):721-726.

45. Becton D, Dahl GV, Ravindranath Y, et al. Randomized use of cyclosporin A (CsA) to modulate P-glycoprotein in children with AML in remission: Pediatric Oncology Group Study 9421. Blood 2006:107(4):1315-1324.

46. Creutzig U, Zimmermann M, Lehrnbecher T, et al. Less toxicity by optimizing chemotherapy, but not by addition of granulocyte colony-stimulating factor in children and adolescents with acute myeloid leukemia: Results of AML-BFM 98. Journal of Clinical Oncology 2006:24(27):4499-4506.

47. Gaynon PS, Harris RE, Altman AJ, et al. Bone marrow transplantation versus prolonged intensive chemotherapy for children with acute lymphoblastic leukemia and an initial bone marrow relapse within 12 months of the completion of primary therapy: Children's Oncology Group study CCG-1941. Journal of Clinical Oncology 2006:24(19):3150-3156.

48. Kung FH, Schwartz CL, Ferree CR, et al. POG 8625: a randomized trial comparing chemotherapy with chemoradiotherapy for children and adolescents with Stages I, IIA, IIIA1 Hodgkin Disease: a report from the Children's Oncology Group. Journal of Pediatric Hematology/Oncology 2006:28(6):362-368.

49. Matloub Y, Lindemulder S, Gaynon PS, et al. Intrathecal triple therapy decreases central nervous system relapse but fails to improve event-free survival when compared with intrathecal methotrexate: results of the Children's Cancer Group (CCG) 1952 study for standard-risk acute lymphoblastic leukemia, reported by the Children's Oncology Group. Blood 2006:108(4):1165-1173.

50. Mitchell C, Pritchard-Jones K, Shannon R, et al. Immediate nephrectomy versus preoperative chemotherapy in the management of non-metastatic Wilms' tumour: results of a randomised trial (UKW3) by the UK Children's Cancer Study Group. European journal of cancer 2006:42(15):2554-2562.

51. Packer RJ, Gajjar A, Vezina G, et al. Phase III study of craniospinal radiation therapy followed by adjuvant chemotherapy for newly diagnosed average-risk medulloblastoma. Journal of Clinical Oncology 2006:24(25):4202-4208.

52. Conter V, Valsecchi MG, Silvestri D, et al. Pulses of vincristine and dexamethasone in addition to intensive chemotherapy for children with intermediate-risk acute lymphoblastic leukaemia: a multicentre randomised trial. Lancet 2007:369(9556):123-131.

53. Le Deley MC, Guinebretiere JM, Gentet JC, et al. SFOP OS94: A randomised trial comparing preoperative high-dose methotrexate plus doxorubicin to high-dose methotrexate plus etoposide and ifosfamide in osteosarcoma patients. European journal of cancer 2007:43(4):752-761.

54. Patte C, Auperin A, Gerrard M, et al. Results of the randomized international FAB/LMB96 trial for intermediate risk B-cell non-Hodgkin lymphoma in children and adolescents: it is possible to reduce treatment for the early responding patients. Blood 2007:109(7):2773-2780.

55. Karachunskiy A, Herold R, von Stackelberg A, et al. Results of the first randomized multicentre trial on childhood acute lymphoblastic leukaemia in Russia. Leukemia 2008:22(6):1144-1153.

56. Pearson ADJ, Pinkerton CR, Lewis IJ, et al. High-dose rapid and standard induction chemotherapy for patients aged over 1 year with stage 4 neuroblastoma: a randomised trial. Lancet Oncology 2008:9(3):247-256.

57. Von Stackelberg A, Hartmann R, Buhrer C, et al. High-dose compared with intermediate-dose methotrexate in children with a first relapse of acute lymphoblastic leukemia. Blood 2008:111(5):2573-2580.

58. Arndt CAS, Stoner JA, Hawkins DS, et al. Vincristine, actinomycin, and cyclophosphamide compared with vincristine, actinomycin, and cyclophosphamide alternating with vincristine, topotecan, and cyclophosphamide for intermediate-risk rhabdomyosarcoma: Children's Oncology Group Study D9803. Journal of Clinical Oncology 2009:27(31):5182-5188.

59. Brandalise SR, Pinheiro VR, Aguiar SS, et al. Benefits of the intermittent use of 6-mercaptopurine and methotrexate in maintenance treatment for low-risk acute lymphoblastic leukemia in children: randomized trial from the Brazilian Childhood Cooperative Group--protocol ALL-99. Journal of Clinical Oncology 2010:28(11):1911-1918.

60. Le Deley M-C, Rosolen A, Williams DM, et al. Vinblastine in children and adolescents with high-risk anaplastic large-cell lymphoma: results of the randomized ALCL99-vinblastine trial. Journal of Clinical Oncology 2010:28(25):3987-3993.

61. Nagatoshi Y, Matsuzaki A, Suminoe A, et al. Randomized trial to compare LSA2L2-type maintenance therapy to daily 6-mercaptopurine and weekly methotrexate with vincristine and dexamethasone pulse for children with acute lymphoblastic leukemia. Pediatric Blood & Cancer 2010:55(2):239-247.

62. Lange BJ, Yang RK, Gan J, et al. Soluble interleukin-2 receptor alpha activation in a Children's Oncology Group randomized trial of interleukin-2 therapy for pediatric acute myeloid leukemia. Pediatric Blood & Cancer 2011:57(3):398-405.

63. Ater JL, Zhou T, Holmes E, et al. Randomized study of two chemotherapy regimens for treatment of low-grade glioma in young children: A report from the Children's Oncology Group. Journal of Clinical Oncology 2012:30(21):2641-2647.

64. Hasle H, Abrahamsson J, Forestier E, et al. Gemtuzumab ozogamicin as postconsolidation therapy does not prevent relapse in children with AML: Results from NOPHO-AML 2004. Blood 2012:120(5):978-984.

65. Oberlin O, Rey A, Sanchez de Toledo J, et al. Randomized comparison of intensified six-drug versus standard three-drug chemotherapy for high-risk nonmetastatic rhabdomyosarcoma and other chemotherapy-sensitive childhood soft tissue sarcomas: long-term results from the International Society of Pediatric Oncology MMT95 study. Journal of Clinical Oncology 2012:30(20):2457-2465.

66. Creutzig U, Zimmermann M, Bourquin J-P, et al. Randomized trial comparing liposomal daunorubicin with idarubicin as induction for pediatric acute myeloid leukemia: results from Study AML-BFM 2004. Blood 2013:122(1):37-43.

67. Kaspers GJL, Zimmermann M, Reinhardt D, et al. Improved outcome in pediatric relapsed acute myeloid leukemia: Results of a randomized trial on liposomal daunorubicin by the international BFM study group. Journal of Clinical Oncology 2013:31(5):599-607.

68. Tarbell NJ, Friedman H, Polkinghorn WR, et al. High-risk medulloblastoma: a pediatric oncology group randomized trial of chemotherapy before or after radiation therapy (POG 9031). Journal of Clinical Oncology 2013:31(23):2936-2941.

69. Termuhlen AM, Smith LM, Perkins SL, et al. Disseminated lymphoblastic lymphoma in children and adolescents: results of the COG A5971 trial: a report from the Children's Oncology Group. British journal of haematology 2013:162(6):792-801.

70. Vora A, Goulden N, Wade R, et al. Treatment reduction for children and young adults with low-risk acute lymphoblastic leukaemia defined by minimal residual disease (UKALL 2003): a randomised controlled trial. Lancet Oncology 2013:14(3):199-209.

71. Friedman DL, Chen L, Wolden S, et al. Dose-intensive response-based chemotherapy and radiation therapy for children and adolescents with newly diagnosed intermediate-risk hodgkin lymphoma: a report from the Children's Oncology Group Study AHOD0031. Journal of Clinical Oncology 2014:32(32):3651-3658.

72. Strother DR, Lafay-Cousin L, Boyett JM, et al. Benefit from prolonged dose-intensive chemotherapy for infants with malignant brain tumors is restricted to patients with ependymoma: a report of the Pediatric Oncology Group randomized controlled trial 9233/34. Neuro-oncology 2014:16(3):457-465.

73. Vora A, Goulden N, Mitchell C, et al. Augmented post-remission therapy for a minimal residual disease-defined high-risk subgroup of children and young people with clinical standard-risk and intermediate-risk acute lymphoblastic leukaemia (UKALL 2003): a randomised controlled trial. Lancet Oncology 2014:15(8):809-818.

74. Wagner JE, Eapen M, Carter S, et al. One-unit versus two-unit cord-blood transplantation for hematologic cancers. New England Journal of Medicine 2014:371(18):1685-1694.

75. Zaghloul MS, Eldebawy E, Ahmed S, et al. Hypofractionated conformal radiotherapy for pediatric diffuse intrinsic pontine glioma (DIPG): a randomized controlled trial. Radiotherapy & Oncology 2014:111(1):35-40.

76. Creutzig U, Dworzak M, Zimmermann M, et al. Randomised introduction of 2-CDA as intensification during consolidation for children with high-risk AML - Results from study AML-BFM 2004. Klinische Padiatrie 2015:227(3):116-122.

77. Pritchard-Jones K, Bergeron C, de Camargo B, et al. Omission of doxorubicin from the treatment of stage II-III, intermediate-risk Wilms' tumour (SIOP WT 2001): an open-label, non-inferiority, randomised controlled trial. Lancet 2015:386(9999):1156-1164.

**List of Excluded Studies:**

1. Karon M, Freireich EJ, Frei E, et al. The role of vincristine in the treatment of childhood acute leukemia. Clinical pharmacology and therapeutics 1966:7(3):332-339.

2. Wolff JA, Newton WA, Jr., Krivit W, et al. Single versus multiple dose dactinomycin therapy of Wilms's tumor. A controlled co-operative study conducted by the Children's Cancer Study Group A (formerly Acute Leukemia Co-operative Chemotherapy Group A). New England Journal of Medicine 1968:279(6):290-294.

3. Aur RJ, Simone JV, Hustu HO, et al. A comparative study of central nervous system irradiation and intensive chemotherapy early in remission of childhood acute lymphocytic leukemia. Cancer 1972:29(2):381-391.

4. Wolff JA, D'Angio G, Hartmann J, et al. Long-term evaluation of single versus multiple courses of actinomycin D therapy of Wilm's tumor. The New England journal of medicine 1974:290(2):84-86.

5. Fernbach DJ, George SL, Sutow WW, et al. Long-term results of reinforcement therapy in children with acute leukemia. Cancer 1975:36(5):1552-1559.

6. Fujimoto T, Goya H, Nakagawa K. Comparison of high dose infusion of methotrexate (MTX) vs sequential complementary method for maintenance of remission in acute childhood leukemia. A cooperative study. Proceedings of the American Association for Cancer Research 1975:16(66):no.257.

7. Rivera G, Avery T, Pratt C. 4' Demethylepipodophyllotoxin 9 (4,6 O 2 thenylidene beta D glucopyranoside) (NSC 122819; VM 26) and 4' demethylepipodophyllotoxin 9 (4,6 O ethylidene beta D glucopyranoside) (NSC 141540; VP 16 213) in childhood cancer: preliminary observations. CANCER CHEMOTHERREP 1975:59(4):743-749.

8. Lemerle J, Voute PA, Tournade MF, et al. Preoperative versus postoperative radiotherapy, single versus multiple courses of actinomycin D, in the treatment of Wilms' tumor. Preliminary results of a controlled clinical trial conducted by the International Society of Paediatric Oncology (S.I.O.P.). Cancer 1976:38(2):647-654.

9. Treatment of acute lymphoblastic leukaemia: effect of variation in length of treatment on duration of remission. Report to the Medical Research Council by the Working Party on Leukaemia in Childhood. British medical journal 1977:2(6085):495-497.

10. Randomized trial of adjuvant chemotherapy in osteogenic osteosarcoma: comparison of altering sequential administrations of high doses of adriamycin, methotrexate, and cyclophosphamide with a 6-month administration of high-dose adriamycin followed by a low-dose semicontinuous chemotherapy. EORTC Osteosarcoma Working Party Group. Recent results in cancer researchFortschritte der KrebsforschungProgres dans les recherches sur le cancer 1978:68:28-32.

11. Aur RJ, Simone JV, Verzosa MS, et al. Childhood acute lymphocytic leukemia: study VIII. Cancer 1978:42(5):2123-2134.

12. Jones PHM, Pearson D, Johnson AL. Management of nephroblastoma in childhood. Clinical study of two forms of maintenance chemotherapy. Archives of Disease in Childhood 1978:53(2):112-119.

13. Rivera G, Murphy SB, Aur RJA. Recurrent childhood lymphocytic leukemia. Clinical and cytokinetic studies of cytosine arabinoside and methotrexate for maintenance of second hematologic remission. Cancer 1978:42(6):2521-2528.

14. Baum E, Sather H, Nachman J. Relapse rates following cessation of chemotherapy during complete remission of acute lymphocytic leukemia. A report from Children's Cancer Study Group. Medical and pediatric oncology 1979:7(1):25-34.

15. Doering EJ, Nitschke R, Haggard ME. Phase II study demonstrating failure of both a five-drug continuous-therapy regimen and a two-drug pulse-therapy regimen in the treatment of metastatic neuroblastoma: Southwest Oncology Group Study 822. Cancer treatment reports 1979:63(8):1383-1384.

16. Ferrant A, Hulhoven R, Bosly A, et al. Clinical trials with daunorubicin-DNA and adriamycin-DNA in acute lymphoblastic leukemia of childhood, acute nonlymphoblastic leukemia, and bronchogenic carcinoma. Cancer Chemotherapy & Pharmacology 1979:2(1):67-71.

17. Rausen AR, Glidewell O, Cuttner J. Superiority of L-asparaginase combination chemotherapy in advanced acute lymphocytic leukemia of childhood. Randomized comparative trial of combination versus solo therapy. Cancer clinical trials 1979:2(2):137-144.

18. Camitta BM, Pinkel D, Thatcher LG. Failure of early intensive chemotherapy to improve prognosis in childhood acute lymphocytic leukemia. Medical and pediatric oncology 1980:8(4):383-389.

19. Ekert H, Waters KD, Matthews RN. A randomized study of intermittent chemotherapy with or without BCG inoculation in maintenance therapy of childhood ALL. Medical and pediatric oncology 1980:8(4):353-360.

20. Jacquillat C, Weil M, Auclerc MF. Application of the study of prognostic factors to the treatment of childhood (<20 years old) acute lymphoblastic leukemia. Protocol 08 LA 74. Bulletin du cancer 1980:67(4):458-469.

21. Murphy SB, Hustu HO. A randomized trial of combined modality therapy of childhood non-Hodgkin's lymphoma. Cancer 1980:45(4):630-637.

22. Anderson J, Krivit W, Chilcote R, et al. Comparison of the therapeutic response of patients with childhood acute lymphoblastic leukemia in relapse to vindesine versus vincristine in combination with prednisone and L-asparaginase: a phase III trial. Cancer treatment reports 1981:65(11-12):1015-1019.

23. D'Angio GJ, Evans A, Breslow N. The treatment of Wilms' tumor: Results of the second National Wilms' Tumor Study. Cancer 1981:47(9):2302-2311.

24. D'Angio GJ, Littman P, Nesbit M. Evaluation of radiation therapy factors in prophylactic central nervous system irradiation for childhood leukemia: A report from the children's cancer study group. International Journal of Radiation Oncology Biology Physics 1981:7(8):1031-1038.

25. Nesbit ME, Jr., Sather HN, Robison LL, et al. Presymptomatic central nervous system therapy in previously untreated childhood acute lymphoblastic leukaemia: comparison of 1800 rad and 2400 rad. A report for Children's Cancer Study Group. Lancet 1981:1(8218):461-466.

26. SackmannMuriel F, Morgenfeld M, Kvicala R. Hodgkin's disease in childhood. Therapy results in Argentina. American Journal of Pediatric Hematology/Oncology 1981:3(3):247-254.

27. Sexauer CL, Vietti T, Humphrey GB. Combination chemotherapy study for remission maintenance in ALL: An evaluation of vincristine, cyclophosphamide and vincristine, cyclophosphamide, and BCNU. A Southwest oncology group phase II study. American Journal of Pediatric Hematology/Oncology 1981:3(3):255-257.

28. Van Eys J, Chen T, Moore T. Adjuvant chemotherapy for medulloblastoma and ependymoma using Iv vincristine, intrathecal methotrexate, and intrathecal hydrocortisone: A southwest oncology group study. Cancer treatment reports 1981:65(7-8):681-684.

29. The treatment of acute lymphoblastic leukaemia (ALL) in childhood, UKALL III: the effects of added cytosine arabinoside and/or asparaginase, and a comparison of continuous or discontinuous mercaptopurine in regimens for standard risk ALL. Medical and pediatric oncology 1982:10(5):501-510.

30. Duration of chemotherapy in childhood acute lymphoblastic leukaemia. The Medical Research Council's Working Party on Leukaemia in Childhood. Medical & Pediatric Oncology 1982:10(5):511-520.

31. Nesbit M, Sather H, Robison L. The duration of chemotherapy for childhood acute lymphoblastic leukemia (ALL): A randomized study of 316 patients. Proceedings of the American Society of Clinical OncologyVol 1982:1:480.

32. Nesbit ME, Sather H, Robison LL, et al. Sanctuary therapy: a randomized trial of 724 children with previously untreated acute lymphoblastic leukemia: A Report from Children's Cancer Study Group. Cancer research 1982:42(2):674-680.

33. Sullivan MP, Chen T, Dyment PG, et al. Equivalence of intrathecal chemotherapy and radiotherapy as central nervous system prophylaxis in children with acute lymphatic leukemia: a pediatric oncology group study. Blood 1982:60(4):948-958.

34. Sullivan MP, Fuller LM, Chen T. Intergroup Hodgkin's disease in children study of stages I and II: A preliminary report. Cancer treatment reports 1982:66(4):937-947.

35. Anderson JR, Wilson JF, Jenkin DT, et al. Childhood non-Hodgkin's lymphoma. The results of a randomized therapeutic trial comparing a 4-drug regimen (COMP) with a 10-drug regimen (LSA2-L2). New England Journal of Medicine 1983:308(10):559-565.

36. Freeman AI, Weinberg V, Brecher ML, et al. Comparison of intermediate-dose methotrexate with cranial irradiation for the post-induction treatment of acute lymphocytic leukemia in children. New England Journal of Medicine 1983:308(9):477-484.

37. Lemerle J, Voute PA, Tournade MF, et al. Effectiveness of preoperative chemotherapy in Wilms' tumor: results of an International Society of Paediatric Oncology (SIOP) clinical trial. Journal of Clinical Oncology 1983:1(10):604-609.

38. Nesbit ME, Jr., Sather HN, Robison LL, et al. Randomized study of 3 years versus 5 years of chemotherapy in childhood acute lymphoblastic leukemia. Journal of Clinical Oncology 1983:1(5):308-316.

39. Sallan SE, Hitchcock Bryan S, Gelber R. Influence of intensive asparaginase in the treatment of childhood non-T-cell acute lymphoblastic leukemia. Cancer research 1983:43(11):5601-5607.

40. Cangir A, Ragab AH, Steuber P. Combination chemotherapy with vincristine (NSC-67574), procarbazine (NSC-77213), prednisone (NSC-10023) with or without nitrogen mustard (NSC-762)(MOPP vs OPP) in children with recurrent brain tumors. Medical and pediatric oncology 1984:12(1):1-3.

41. Evans WE, Crom WR, Stewart CF, et al. Methotrexate systemic clearance influences probability of relapse in children with standard-risk acute lymphocytic leukaemia. Lancet 1984:1(8373):359-362.

42. Krischer J, Land VJ, Civin CI, et al. Evaluation of AMSA in children with acute leukemia. A Pediatric Oncology Group study. Cancer 1984:54(2):207-210.

43. Lilleyman JS, Campbell RHA. Vindesine in relapsed childhood ALL. A pilot study by the United Kingdom children's cancer study group. European Paediatric Haematology and Oncology 1984:1(1):37-38.

44. Mott MG, Eden OB, Palmer MK. Adjuvant low dose radiation in childhood non-Hodgkin's lymphoma. (Report from the United Kingdom Childrens' Cancer Study Group - UKCCSG). British journal of cancer 1984:50(4):463-469.

45. Movassaghi N, Higgins G, Pyesmany A. Evaluation of cyclocytidine in reinduction and maintenance therapy of children with acute nonlymphocytic leukemia previously treated with cytosine arabinoside: A report from children's cancer study group. Medical and pediatric oncology 1984:12(5):352-356.

46. Pui CH, Aur RJA, Bowman WP. Failure of late intensification therapy to improve a poor result in childhood lymphoblastic leukemia. Cancer research 1984:44(8):3593-3598.

47. Sackmann Muriel F, Svarch E, Pavlovsky S. Alternating pulses of vincristine-prednisone with cytarabine-cyclophosphamide versus vincristine-prednisone in the maintenance therapy of acute lymphoblastic leukemia. Cancer treatment reports 1984:68(4):581-586.

48. Flamant F, Rodary C, Voute PA, et al. Primary chemotherapy in the treatment of rhabdomyosarcoma in children: Trial of the international society of pediatric oncology (SIOP) preliminary results. Radiotherapy and Oncology 1985:3(3):227-236.

49. Land VJ, Thomas PR, Boyett JM, et al. Comparison of maintenance treatment regimens for first central nervous system relapse in children with acute lymphocytic leukemia. A Pediatric Oncology Group study. Cancer 1985:56(1):81-87.

50. Mehta P, Gardner R, GrahamPole J, et al. Methylprednisolone is effective in chemotherapy-induced emesis: Results of a double blind randomized trial in children. Proceedings of the American Association for Cancer ResearchVOL 1985:26:No. 602.

51. Brecher ML, Weinberg V, Boyett JM, et al. Intermediate dose methotrexate in childhood acute lymphoblastic leukemia resulting in decreased incidence of testicular relapse. Cancer 1986:58(5):1024-1028.

52. Chessells JM, Durrant J, Hardy RM, et al. Medical Research Council leukaemia trial--UKALL V: an attempt to reduce the immunosuppressive effects of therapy in childhood acute lymphoblastic leukemia. Report to the Council by the Working Party on Leukaemia in Childhood. Journal of Clinical Oncology 1986:4(12):1758-1764.

53. Link MP, Goorin AM, Miser AW. The effect of adjuvant chemotherapy on relapse-free survival in patients with osteosarcoma of the extremity. New England Journal of Medicine 1986:314(25):1600-1606.

54. Ragab AH, Boyett JM, Frankel L, et al. Rubidazone in the treatment of recurrent acute leukemia in children. A Pediatric Oncology Group Study. Cancer 1986:57(8):1461-1463.

55. Chan HSL, Correia JA, MacLeod SM. Nabilone versus prochlorperazine for control of cancer chemotherapy-induced emesis in children: A double-blind, crossover trial. Pediatrics 1987:79(6):946-952.

56. Jenkin RD, Boesel C, Ertel I, et al. Brain-stem tumors in childhood: a prospective randomized trial of irradiation with and without adjuvant CCNU, VCR, and prednisone. A report of the Childrens Cancer Study Group. Journal of neurosurgery 1987:66(2):227-233.

57. Krailo M, Ertel I, Makley J, et al. A randomized study comparing high-dose methotrexate with moderate-dose methotrexate as components of adjuvant chemotherapy in childhood nonmetastatic osteosarcoma: a report from the Childrens Cancer Study Group. Medical & Pediatric Oncology 1987:15(2):69-77.

58. Littman P, Coccia P, Bleyer WA, et al. Central nervous system (CNS) prophylaxis in children with low risk acute lymphoblastic leukemia (ALL). International journal of radiation oncology, biology, physics 1987:13(10):1443-1449.

59. Ortega JJ, Javier G, Olive T. Treatment of standard- and high-risk childhood acute lymphoblastic leukaemia with two CNS prophylaxis regimens. Haematology & Blood Transfusion 1987:30:483-492.

60. Zintl F, Plenert W, Malke H. Results of acute lymphoblastic leukemia therapy in childhood with a modified BFM protocol in a multicenter study in the German Democratic Republic. Haematology & Blood Transfusion 1987:30:471-479.

61. Carli M, Pastore G, Perilongo G, et al. Tumor response and toxicity after single high-dose versus standard five-day divided-dose dactinomycin in childhood rhabdomyosarcoma. Journal of Clinical Oncology 1988:6(4):654-658.

62. Gaynon PS, Steinherz PG, Bleyer WA, et al. Intensive therapy for children with acute lymphoblastic leukaemia and unfavourable presenting features. Early conclusions of study CCG-106 by the Childrens Cancer Study Group. Lancet 1988:2(8617):921-924.

63. JankaSchaub GE, Winkler K, Gobel U, et al. Rapidly rotating combination chemotherapy in childhood acute lympoblastic leukemia: Preliminary results of a randomized comparison with conventional treatment. Leukemia 1988:2(12 SUPPL):73s-78s.

64. Koizumi S, Fujimoto T, Takeda T, et al. Comparison of intermittent or continuous methotrexate plus 6-mercaptopurine in regimens for standard-risk acute lymphoblastic leukemia in childhood (JCCLSG-S811). The Japanese Children's Cancer and Leukemia Study Group. Cancer 1988:61(7):1292-1300.

65. Winkler K, Beron G, Delling G, et al. Neoadjuvant chemotherapy of osteosarcoma: Results of a randomized cooperative trial (COSS-82) with salvage chemotherapy based on histological tumor response. Journal of Clinical Oncology 1988:6(2):329-337.

66. Crist W, Boyett J, Jackson J, et al. Prognostic importance of the pre-B-cell immunophenotype and other presenting features in B-lineage childhood acute lymphoblastic leukemia: a Pediatric Oncology Group study. Blood 1989:74(4):1252-1259.

67. Miller DR, Coccia PF, Bleyer WA, et al. Early response to induction therapy as a predictor of disease-free survival and late recurrence of childhood acute lymphoblastic leukemia: a report from the Childrens Cancer Study Group. Journal of Clinical Oncology 1989:7(12):1807-1815.

68. Miller DR, Leikin SL, Albo VC, et al. Three versus five years of maintenance therapy are equivalent in childhood acute lymphoblastic leukemia: a report from the Childrens Cancer Study Group. Journal of Clinical Oncology 1989:7(3):316-325.

69. Sposto R, Ertel IJ, Jenkin RD, et al. The effectiveness of chemotherapy for treatment of high grade astrocytoma in children: results of a randomized trial. A report from the Childrens Cancer Study Group. Journal of neuro-oncology 1989:7(2):165-177.

70. Lampkin BC, Woods WG, Buckley JD, et al. Preliminary results of intensive therapy of children and adolescents with acute nonlymphocytic leukemia--a Childrens Cancer Study Group report. Haematology and blood transfusion 1990:33:210-214.

71. Steuber CP, Culbert SJ, Ravindranath Y, et al. Therapy of childhood acute nonlymphocytic leukemia: the Pediatric Oncology Group experience (1977-1988). Haematology and blood transfusion 1990:33:198-209.

72. Tait DM, Thornton-Jones H, Bloom HJ, et al. Adjuvant chemotherapy for medulloblastoma: the first multi-centre control trial of the International Society of Paediatric Oncology (SIOP I). European journal of cancer 1990:26(4):464-469.

73. Bleyer WA, Sather HN, Nickerson HJ, et al. Monthly pulses of vincristine and prednisone prevent bone marrow and testicular relapse in low-risk childhood acute lymphoblastic leukemia: a report of the CCG-161 study by the Childrens Cancer Study Group. Journal of Clinical Oncology 1991:9(6):1012-1021.

74. Buchanan GR, Boyett JM, Pollock BH, et al. Improved treatment results in boys with overt testicular relapse during or shortly after initial therapy for acute lymphoblastic leukemia. A Pediatric Oncology group study. Cancer 1991:68(1):48-55.

75. Castleberry RP, Kun LE, Shuster JJ, et al. Radiotherapy improves the outlook for patients older than 1 year with Pediatric Oncology Group stage C neuroblastoma. Journal of Clinical Oncology 1991:9(5):789-795.

76. Conner K, Sandler E, Weyman C, et al. Intravenous midazolam versus fentanyl as premedication for painful procedures in pediatric oncology patients. Journal of Pediatric Oncology Nursing 1991:8(2):86-87.

77. Culbert SJ, Shuster JJ, Land VJ, et al. Remission induction and continuation therapy in children with their first relapse of acute lymphoid leukemia. A Pediatric Oncology Group study. Cancer 1991:67(1):37-42.

78. De Camargo B, Franco EL. Single-dose versus fractionated-dose dactinomycin in the treatment of Wilms' tumor: Preliminary results of a clinical trial. Cancer 1991:67(12):2990-2996.

79. Eden OB, Lilleyman JS, Richards S, et al. Results of Medical Research Council Childhood Leukaemia Trial UKALL VIII (report to the Medical Research Council on behalf of the Working Party on Leukaemia in Childhood). British journal of haematology 1991:78(2):187-196.

80. Henze G, Fengler R, Hartmann R, et al. Six-year experience with a comprehensive approach to the treatment of recurrent childhood acute lymphoblastic leukemia (ALL-REZ BFM 85). A relapse study of the BFM group. Blood 1991:78(5):1166-1172.

81. Krischer JP, Ragab AH, Kun L, et al. Nitrogen mustard, vincristine, procarbazine, and prednisone as adjuvant chemotherapy in the treatment of medulloblastoma. A Pediatric Oncology Group study. Journal of neurosurgery 1991:74(6):905-909.

82. Rivera GK, Raimondi SC, Hancock ML, et al. Improved outcome in childhood acute lymphoblastic leukaemia with reinforced early treatment and rotational combination chemotherapy. Lancet 1991:337(8733):61-66.

83. Sullivan MP, Brecher M, Ramirez I, et al. High-dose cyclophosphamide-high-dose methotrexate with coordinated intrathecal therapy for advanced nonlymphoblastic lymphoma of childhood: results of a Pediatric Oncology Group study. American Journal of Pediatric Hematology/Oncology 1991:13(3):288-295.

84. Sullivan MP, Fuller LM, Berard C, et al. Comparative effectiveness of two combined modality regimens in the treatment of surgical stage III Hodgkin's disease in children. An 8-year follow-up study by the Pediatric Oncology Group. American Journal of Pediatric Hematology/Oncology 1991:13(4):450-458.

85. Tsuchida M, Akatsuka J, Bessho F, et al. Treatment of acute lymphoblastic leukemia in the Tokyo Children's Cancer Study Group--preliminary results of L84-11 protocol. Acta Paediatrica Japonica 1991:33(4):522-532.

86. Weiner MA, Leventhal BG, Marcus R, et al. Intensive chemotherapy and low-dose radiotherapy for the treatment of advanced-stage Hodgkin's disease in pediatric patients: A Pediatric Oncology Group study. Journal of Clinical Oncology 1991:9(9):1591-1598.

87. Crist W, Shuster J, Look T, et al. Current results of studies of immunophenotype-, age- and leukocyte-based therapy for children with acute lymphoblastic leukemia. Leukemia 1992:6(SUPPL. 2):162-166.

88. Harris MB, Shuster JJ, Carroll A, et al. Trisomy of leukemic cell chromosomes 4 and 10 identifies children with B- progenitor cell acute lymphoblastic leukemia with a very low risk of treatment failure: A Pediatric Oncology Group Study. Blood 1992:79(12):3316-3324.

89. Miser JS, Roloff J, Blatt J, et al. Lack of significant activity of 2'-deoxycoformycin alone or in combination with adenine arabinoside in relapsed childhood acute lymphoblastic leukemia. A randomized phase II trial from the Childrens Cancer Study Group. American Journal of Clinical Oncology 1992:15(6):490-493.

90. Pui CH, Simone JV, Hancock ML, et al. Impact of three methods of treatment intensification on acute lymphoblastic leukemia in children: long-term results of St Jude total therapy study X. Leukemia 1992:6(2):150-157.

91. Amadori S, Testi AM, Arico M, et al. Prospective comparative study of bone marrow transplantation and postremission chemotherapy for childhood acute myelogenous leukemia. The Associazione Italiana Ematologia ed Oncologia Pediatrica Cooperative Group. Journal of Clinical Oncology 1993:11(6):1046-1054.

92. Cherlow JM, Steinherz PG, Sather HN, et al. The role of radiation therapy in the treatment of acute lymphoblastic leukemia with lymphomatous presentation: a report from the Childrens Cancer Group. International journal of radiation oncology, biology, physics 1993:27(5):1001-1009.

93. Creutzig U, Ritter J, Zimmermann M, et al. Does cranial irradiation reduce the risk for bone marrow relapse in acute myelogenous leukemia? Unexpected results of the Childhood Acute Myelogenous Leukemia Study BFM-87. Journal of Clinical Oncology 1993:11(2):279-286.

94. Gaynon PS, Steinherz PG, Bleyer WA, et al. Improved therapy for children with acute lymphoblastic leukemia and unfavorable presenting features: a follow-up report of the Childrens Cancer Group Study CCG-106. Journal of Clinical Oncology 1993:11(11):2234-2242.

95. Miser JS, Pritchard DJ, Rock MG, et al. Osteosarcoma in adolescents and young adults: new developments and controversies. The Mayo Clinic studies. Cancer treatment and research 1993:62:333-338.

96. Sadowitz PD, Smith SD, Shuster J, et al. Treatment of late bone marrow relapse in children with acute lymphoblastic leukemia: a Pediatric Oncology Group study. Blood 1993:81(3):602-609.

97. Tsukada M, Komiyama A, Nakazawa S, et al. Treatment of standard risk acute lymphoblastic leukemia in children with the Tokyo Children Cancer Study Group (TCCSG) L84-11 protocol in Japan. International journal of hematology 1993:57(1):1-7.

98. Tubergen DG, Gilchrist GS, O'Brien RT, et al. Prevention of CNS disease in intermediate-risk acute lymphoblastic leukemia: comparison of cranial radiation and intrathecal methotrexate and the importance of systemic therapy: a Childrens Cancer Group report. Journal of Clinical Oncology 1993:11(3):520-526.

99. Winick NJ, Smith SD, Shuster J, et al. Treatment of CNS relapse in children with acute lymphoblastic leukemia: A Pediatric Oncology Group study. Journal of Clinical Oncology 1993:11(2):271-278.

100. Yang CP, Lin ST, Liang DC, et al. Treatment of childhood acute lymphoblastic leukemia with protocol TCL-842 in Taiwan: the Taiwan Children's Cancer Study Group. Journal of the Formosan Medical Association 1993:92(5):431-439.

101. Castleberry RP, Cantor AB, Green AA, et al. Phase II investigational window using carboplatin, iproplatin, ifosfamide, and epirubicin in children with untreated disseminated neuroblastoma: a Pediatric Oncology Group study. Journal of Clinical Oncology 1994:12(8):1616-1620.

102. Creutzig U, Ritter J, Zimmermann M, et al. Superior results by cranial irradiation in children with acute myelogenous leukemia: An update of study AML-BFM-87. Onkologie 1994:17(1):66-68.

103. De Camargo B, Franco EL. A randomized clinical trial of single-dose versus fractionated-dose dactinomycin in the treatment of Wilms' tumor: Results after extended follow- up. Cancer 1994:73(12):3081-3086.

104. Elder JS. Results of the Sixth International Society of Pediatric Oncology Wilms' tumor trial and study: a risk-adapted therapeutic approach in Wilms' tumor. The Journal of urology 1994:152(1):271-272.

105. Gilchrist GS, Tubergen DG, Sather HN, et al. Low numbers of CSF blasts at diagnosis do not predict for the development of CNS leukemia in children with intermediate-risk acute lymphoblastic leukemia: A Childrens Cancer Group report. Journal of Clinical Oncology 1994:12(12):2594-2600.

106. Koizumi S, Fujimoto T. Improvement in treatment of childhood acute lymphoblastic leukemia: a 10-year study by the Children's Cancer and Leukemia Study Group. International journal of hematology 1994:59(2):99-112.

107. Land VJ, Shuster JJ, Crist WM, et al. Comparison of two schedules of intermediate-dose methotrexate and cytarabine consolidation therapy for childhood B-precursor cell acute lymphoblastic leukemia: a Pediatric Oncology Group study. Journal of Clinical Oncology 1994:12(9):1939-1945.

108. Nesbit ME, Jr., Buckley JD, Feig SA, et al. Chemotherapy for induction of remission of childhood acute myeloid leukemia followed by marrow transplantation or multiagent chemotherapy: a report from the Childrens Cancer Group. Journal of Clinical Oncology 1994:12(1):127-135.

109. Sertoli MR, Santini G, Chisesi T, et al. MACOP-B versus ProMACE-MOPP in the treatment of advanced diffuse non- Hodgkin's lymphoma: Results of a prospective randomized trial by the Non- Hodgkin's Lymphoma Cooperative Study Group. Journal of Clinical Oncology 1994:12(7):1366-1374.

110. Wells RJ, Woods WG, Buckley JD, et al. Treatment of newly diagnosed children and adolescents with acute myeloid leukemia: A Childrens Cancer Group study. Journal of Clinical Oncology 1994:12(11):2367-2377.

111. Bailey CC, Gnekow A, Wellek S, et al. Prospective randomised trial of chemotherapy given before radiotherapy in childhood medulloblastoma. International Society of Paediatric Oncology (SIOP) and the (German) Society of Paediatric Oncology (GPO): SIOP II. Medical & Pediatric Oncology 1995:25(3):166-178.

112. Chessells JM, Bailey C, Richards SM. Intensification of treatment and survival in all children with lymphoblastic leukaemia: results of UK Medical Research Council trial UKALL X. Medical Research Council Working Party on Childhood Leukaemia. Lancet 1995:345(8943):143-148.

113. Cohen BH, Zeltzer PM, Boyett JM, et al. Prognostic factors and treatment results for supratentorial primitive neuroectodermal tumors in children using radiation and chemotherapy: a Childrens Cancer Group randomized trial. Journal of Clinical Oncology 1995:13(7):1687-1696.

114. Crist W, Gehan EA, Ragab AH, et al. The Third Intergroup Rhabdomyosarcoma Study. Journal of Clinical Oncology 1995:13(3):610-630.

115. Donaldson SS, Asmar L, Breneman J, et al. Hyperfractionated radiation in children with rhabdomyosarcoma - Results of an intergroup rhabdomyosarcoma pilot study. International Journal of Radiation Oncology Biology Physics 1995:32(4):903-911.

116. McWilliams NB, Hayes FA, Green AA, et al. Cyclophosphamide/doxorubicin vs. cisplatin/teniposide in the treatment of children older than 12 months of age with disseminated neuroblastoma: a Pediatric Oncology Group Randomized Phase II study. Medical & Pediatric Oncology 1995:24(3):176-180.

117. Sebban C, Browman GP, Lepage E, et al. Prognostic value of early response to chemotherapy assessed by the day 15 bone marrow aspiration in adult acute lymphoblastic leukemia: A prospective analysis of 437 cases and its application for designing induction chemotherapy trials. Leukemia research 1995:19(11):861-868.

118. Tubergen DG, Krailo MD, Meadows AT, et al. Comparison of treatment regimens for pediatric lymphoblastic non-Hodgkin's lymphoma: a Childrens Cancer Group study. Journal of Clinical Oncology 1995:13(6):1368-1376.

119. Calandra T, Gaya H, Zinner SH, et al. Monotherapy with meropenem versus combination therapy with ceftazidime plus amikacin as empiric therapy for fever in granulocytopenic patients with cancer. Antimicrobial Agents and Chemotherapy 1996:40(5):1108-1115.

120. Deutsch M, Thomas PR, Krischer J, et al. Results of a prospective randomized trial comparing standard dose neuraxis irradiation (3,600 cGy/20) with reduced neuraxis irradiation (2,340 cGy/13) in patients with low-stage medulloblastoma. A Combined Children's Cancer Group-Pediatric Oncology Group Study. Pediatric neurosurgery 1996:24(4):167-176.

121. Evans AE, Anderson JR, Lefkowitz-Boudreaux IB, et al. Adjuvant chemotherapy of childhood posterior fossa ependymoma: cranio-spinal irradiation with or without adjuvant CCNU, vincristine, and prednisone: a Childrens Cancer Group study. Medical & Pediatric Oncology 1996:27(1):8-14.

122. Feig SA, Ames MM, Sather HN, et al. Comparison of idarubicin to daunomycin in a randomized multidrug treatment of childhood acute lymphoblastic leukemia at first bone marrow relapse: a report from the Children's Cancer Group. Medical & Pediatric Oncology 1996:27(6):505-514.

123. Richards S, Gray R, Peto R, et al. Duration and intensity of maintenance chemotherapy in acute lymphoblastic leukaemia: Overview of 42 trials involving 12,000 randomised children. Lancet 1996:347(9018):1783-1788.

124. Steuber CP, Krischer J, Holbrook T, et al. Therapy of refractory or recurrent childhood acute myeloid leukemia using amsacrine and etoposide with or without azacitidine: a Pediatric Oncology Group randomized phase II study. Journal of Clinical Oncology 1996:14(5):1521-1525.

125. Brecher ML, Schwenn MR, Coppes MJ, et al. Fractionated cylophosphamide and back to back high dose methotrexate and cytosine arabinoside improves outcome in patients with stage III high grade small non-cleaved cell lymphomas (SNCCL): a randomized trial of the Pediatric Oncology Group. Medical & Pediatric Oncology 1997:29(6):526-533.

126. Conter V, Schrappe M, Arico M, et al. Role of cranial radiotherapy for childhood T-cell acute lymphoblastic leukemia with high WBC count and good response to prednisone. Journal of Clinical Oncology 1997:15(8):2786-2791.

127. Coze C, Hartmann O, Michon J, et al. NB87 induction protocol for stage 4 neuroblastoma in children over 1 year of age: a report from the French Society of Pediatric Oncology. Journal of Clinical Oncology 1997:15(12):3433-3440.

128. Feig SA, Harris RE, Sather HN. Bone marrow transplantation versus chemotherapy for maintenance of second remission of childhood acute lymphoblastic leukemia: A study of the children's cancer group (CCG-1884). Medical and pediatric oncology 1997:29(6):534-540.

129. Freeman AI, Boyett JM, Glicksman AS, et al. Intermediate-dose methotrexate versus cranial irradiation in childhood acute lymphoblastic leukemia: a ten-year follow-up. Medical & Pediatric Oncology 1997:28(2):98-107.

130. Koizumi S, Fujimoto T, Oka T, et al. Overview of clinical studies of childhood acute lymphoblastic leukemia for more than ten years by the Japanese Children's Cancer and Leukemia Study Group. Pediatric hematology and oncology 1997:14(1):17-28.

131. Sackmann-Muriel F, Zubizarreta P, Gallo G, et al. Hodgkin disease in children: results of a prospective randomized trial in a single institution in Argentina. Medical & Pediatric Oncology 1997:29(6):544-552.

132. Souhami RL, Craft AW, Van Der Eijken JW, et al. Randomised trial of two regimens of chemotherapy in operable osteosarcoma: A study of the European Osteosarcoma Intergroup. Lancet 1997:350(9082):911-917.

133. Weiner MA, Leventhal B, Brecher ML, et al. Randomized study of intensive MOPP-ABVD with or without low-dose total-nodal radiation therapy in the treatment of stages IIB, IIIA2, IIIB, and IV Hodgkin's disease in pediatric patients: a Pediatric Oncology Group study. Journal of Clinical Oncology 1997:15(8):2769-2779.

134. Donaldson SS, Torrey M, Link MP, et al. A multidisciplinary study investigating radiotherapy in Ewing's sarcoma: end results of POG #8346. Pediatric Oncology Group. International journal of radiation oncology, biology, physics 1998:42(1):125-135.

135. Hutchinson RJ, Fryer CJ, Davis PC, et al. MOPP or radiation in addition to ABVD in the treatment of pathologically staged advanced Hodgkin's disease in children: results of the Children's Cancer Group Phase III Trial. Journal of Clinical Oncology 1998:16(3):897-906.

136. Kawano Y, Takaue Y, Mimaya J, et al. Marginal benefit/disadvantage of granulocyte colony-stimulating factor therapy after autologous blood stem cell transplantation in children: results of a prospective randomized trial. The Japanese Cooperative Study Group of PBSCT. Blood 1998:92(11):4040-4046.

137. Kuhl J, Muller HL, Berthold F, et al. Preradiation chemotherapy of children and young adults with malignant brain tumors: results of the German pilot trial HIT'88/'89. Klinische Padiatrie 1998:210(4):227-233.

138. Michon JM, Hartmann O, Bouffet E, et al. An open-label, multicentre, randomised phase 2 study of recombinant human granulocyte colony-stimulating factor (filgrastim) as an adjunct to combination chemotherapy in paediatric patients with metastatic neuroblastoma. European journal of cancer 1998:34(7):1063-1069.

139. Nachman J, Sather HN, Cherlow JM, et al. Response of children with high-risk acute lymphoblastic leukemia treated with and without cranial irradiation: a report from the Children's Cancer Group. Journal of Clinical Oncology 1998:16(3):920-930.

140. Nachman JB, Sather HN, Sensel MG, et al. Augmented post-induction therapy for children with high-risk acute lymphoblastic leukemia and a slow response to initial therapy. New England Journal of Medicine 1998:338(23):1663-1671.

141. Richards S, Burrett J, Hann I, et al. Improved survival with early intensification: Combined results from The Medical Research Council childhood ALL randomised trials, UKALL X and UKALL XI. Leukemia 1998:12(7):1031-1036.

142. Schrappe M, Reiter A, Henze G, et al. Prevention of CNS recurrence in childhood ALL: Results with reduced radiotherapy combined with CNS-directed chemotherapy in four consecutive ALL- BFM trials. Klinische Padiatrie 1998:210(4):192-199.

143. Steinherz PG, Gaynon PS, Breneman JC, et al. Treatment of patients with acute lymphoblastic leukemia with bulky extramedullary disease and T-cell phenotype or other poor prognostic features: randomized controlled trial from the Children's Cancer Group. Cancer 1998:82(3):600-612.

144. Amylon MD, Shuster J, Pullen J, et al. Intensive high-dose asparaginase consolidation improves survival for pediatric patients with T cell acute lymphoblastic leukemia and advanced stage lymphoblastic lymphoma: A Pediatric Oncology Group study. Leukemia 1999:13(3):335-342.

145. Asselin BL, Kreissman S, Coppola DJ, et al. Prognostic significance of early response to a single dose of asparaginase in childhood acute lymphoblastic leukemia. Journal of Pediatric Hematology/Oncology 1999:21(1):6-12.

146. Kamps WA, Bokkerink JP, Hahlen K, et al. Intensive treatment of children with acute lymphoblastic leukemia according to ALL-BFM-86 without cranial radiotherapy: results of Dutch Childhood Leukemia Study Group Protocol ALL-7 (1988-1991). Blood 1999:94(4):1226-1236.

147. Kobrinsky NL, Packer RJ, Boyett JM, et al. Etoposide with or without mannitol for the treatment of recurrent or primarily unresponsive brain tumors: a Children's Cancer Group Study, CCG-9881. Journal of neuro-oncology 1999:45(1):47-54.

148. Liang DC, Hung IJ, Yang CP, et al. Unexpected mortality from the use of E. coli L-asparaginase during remission induction therapy for childhood acute lymphoblastic leukemia: a report from the Taiwan Pediatric Oncology Group. Leukemia 1999:13(2):155-160.

149. Mandell LR, Kadota R, Freeman C, et al. There is no role for hyperfractionated radiotherapy in the management of children with newly diagnosed diffuse intrinsic brainstem tumors: results of a Pediatric Oncology Group phase III trial comparing conventional vs. hyperfractionated radiotherapy. International journal of radiation oncology, biology, physics 1999:43(5):959-964.

150. Marina NM, Pappo AS, Parham DM, et al. Chemotherapy dose-intensification for pediatric patients with Ewing's family of tumors and desmoplastic small round-cell tumors: a feasibility study at St. Jude Children's Research Hospital. Journal of Clinical Oncology 1999:17(1):180-190.

151. Matsuzaki A, Okamura J, Ishii E, et al. Treatment of standard-risk acute lymphoblastic leukemia in children: The results of protocol AL841 from the Kyushu-Yamaguchi Children's Cancer Study Group in Japan. Pediatric hematology and oncology 1999:16(3):187-199.

152. Matthay KK, Villablanca JG, Seeger RC, et al. Treatment of high-risk neuroblastoma with intensive chemotherapy, radiotherapy, autologous bone marrow transplantation, and 13-cis-retinoic acid. Children's Cancer Group. New England Journal of Medicine 1999:341(16):1165-1173.

153. Suryanarayan K, Shuster JJ, Donaldson SS, et al. Treatment of localized primary non-Hodgkin's lymphoma of bone in children: A Pediatric Oncology Group Study. Journal of Clinical Oncology 1999:17(2):456-459.

154. Tsurusawa M, Katano N, Yamamoto Y, et al. Improvement in CNS protective treatment in non-high-risk childhood acute lymphoblastic leukemia: report from the Japanese Children's Cancer and Leukemia Study Group. Medical & Pediatric Oncology 1999:32(4):259-256.

155. CoustanSmith E, Sancho J, Hancock ML, et al. Clinical importance of minimal residual disease in childhood acute lymphoblastic leukemia. Blood 2000:96(8):2691-2696.

156. Dahl GV, Lacayo NJ, Brophy N, et al. Mitoxantrone, etoposide, and cyclospine therapy in pediatric patients with recurrent or refractory acute myeloid leukemia. Journal of Clinical Oncology 2000:18(9):1867-1875.

157. Freeman CR, Kepner J, Kun LE, et al. A detrimental effect of a combined chemotherapy-radiotherapy approach in children with diffuse intrinsic brain stem gliomas? International Journal of Radiation Oncology Biology Physics 2000:47(3):561-564.

158. Kohler JA, Imeson J, Ellershaw C, et al. A randomized trial of 13-Cis retinoic acid in children with advanced neuroblastoma after high-dose therapy. British journal of cancer 2000:83(9):1124-1127.

159. Kojima S, Hibi S, Kosaka Y, et al. Immunosuppressive therapy using antithymocyte globulin, cyclosporine, and danazol with or without human granulocyte colony-Stimulating factor in children with acquired aplastic anemia. Blood 2000:96(6):2049-2054.

160. Kortmann RD, Kuhl J, Timmermann B, et al. Postoperative neoadjuvant chemotherapy before radiotherapy as compared to immediate radiotherapy followed by maintenance chemotherapy in the treatment of medulloblastoma in childhood: results of the German prospective randomized trial HIT '91. International journal of radiation oncology, biology, physics 2000:46(2):269-279.

161. Laver JH, Barredo JC, Amylon M, et al. Effects of cranial radiation in children with high risk T cell acute lymphoblastic leukemia: A Pediatric Oncology Group report. Leukemia 2000:14(3):369-373.

162. Loning L, Zimmermann M, Reiter A, et al. Secondary neoplasms subsequent to Berlin-Frankfurt-Munster therapy of acute lymphoblastic leukemia in childhood: Significantly lower risk without cranial radiotherapy. Blood 2000:95(9):2770-2775.

163. Maris JM, Weiss MJ, Guo C, et al. Loss of heterozygosity at 1p36 independently predicts for disease progression but not decreased overall survival probability in neuroblastoma patients: A children's cancer group study. Journal of Clinical Oncology 2000:18(9):1888-1899.

164. Michel G, Landman-Parker J, Auclerc MF, et al. Use of recombinant human granulocyte colony-stimulating factor to increase chemotherapy dose-intensity: a randomized trial in very high-risk childhood acute lymphoblastic leukemia. Journal of Clinical Oncology 2000:18(7):1517-1524.

165. Reiter A, Schrappe M, Ludwig WD, et al. Intensive ALL-type therapy without local radiotherapy provides a 90% event-free survival for children with T-cell lymphoblastic lymphoma: A BFM Group report. Blood 2000:95(2):416-421.

166. Shamberger RC, Laquaglia MP, Krailo MD, et al. Ewing sarcoma of the rib: results of an intergroup study with analysis of outcome by timing of resection. Journal of Thoracic & Cardiovascular Surgery 2000:119(6):1154-1161.

167. Sievers EL, Lange BJ, Sondel PM, et al. Children's cancer group trials of interleukin-2 therapy to prevent relapse of acute myelogenous leukemia. The cancer journal from Scientific American 2000:6(Suppl 1):S39-44.

168. Vilmer E, Suciu S, Ferster A, et al. Long-term results of three randomized trials (58831, 58832, 58881) in childhood acute lymphoblastic leukemia: A CLCG-EORTC report. Leukemia 2000:14(12):2257-2266.

169. Wells RJ, Woods WG, Buckley JD, et al. Therapy for acute myeloid leukemia: intensive timing of induction chemotherapy. Current oncology reports 2000:2(6):524-528.

170. Breitfeld PP, Lyden E, Raney RB, et al. Ifosfamide and etoposide are superior to vincristine and melphalan for pediatric metastatic rhabdomyosarcoma when administered with irradiation and combination chemotherapy: A report from the Intergroup Rhabdomyosarcoma Study Group. Journal of Pediatric Hematology/Oncology 2001:23(4):225-233.

171. Goldman SC, Holcenberg JS, Finklestein JZ, et al. A randomized comparison between rasburicase and allopurinol in children with lymphoma or leukemia at high risk for tumor lysis. Blood 2001:97(10):2998-3003.

172. Lauer SJ, Shuster JJ, Mahoney Jr DH, et al. A comparison of early intensive methotrexate/mercaptopurine with early intensive alternating combination chemotherapy for high-risk B-precursor acute lymphoblastic leukemia: A Pediatric Oncology Group phase III randomized trial. Leukemia 2001:15(7):1038-1045.

173. Laver JH, Mahmoud H, Pick TE, et al. Results of a randomized phase III trial in children and adolescents with advanced stage diffuse large cell non Hodgkin's lymphoma: a Pediatric Oncology Group study. Leukemia & lymphoma 2001:42(3):399-405.

174. Manabe A, Tsuchida M, Hanada R, et al. Delay of the diagnostic lumbar puncture and intrathecal chemotherapy in children with acute lymphoblastic leukemia who undergo routine corticosteroid testing: Tokyo Children's Cancer Study Group Study L89-12. Journal of Clinical Oncology 2001:19(13):3182-3187.

175. Ortega JJ, Ribera JM, Oriol A, et al. Early and delayed consolidation chemotherapy significantly improves the outcome of children with intermediate-risk acute lymphoblastic leukemia. Final results of the prospective randomized PETHEMA ALL-89 TRIAL. Haematologica 2001:86(6):586-595.

176. Rescorla F, Billmire D, Stolar C, et al. The effect of cisplatin dose and surgical resection in children with malignant germ cell tumors at the sacrococcygeal region: A pediatric intergroup trial (POG 9049/CCG 8882). Journal of pediatric surgery 2001:36(1):12-17.

177. Woods WG, Neudorf S, Gold S, et al. A comparison of allogeneic bone marrow transplantation, autologous bone marrow transplantation, and aggressive chemotherapy in children with acute myeloid leukemia in remission: A report from the Children's Cancer Group. Blood 2001:97(1):56-62.

178. Avramis VI, Sencer S, Periclou AP, et al. A randomized comparison of native Escherichia coli asparaginase and polyethylene glycol conjugated asparaginase for treatment of children with newly diagnosed standard-risk acute lymphoblastic leukemia: a Children's Cancer Group study. Blood 2002:99(6):1986-1994.

179. Chessells JM, Harrison G, Richards SM, et al. Failure of a new protocol to improve treatment results in paediatric lymphoblastic leukaemia: Lessons from the UK Medical Research Council trials UKALL X and UKALL XI. British journal of haematology 2002:118(2):445-455.

180. Couban S, Simpson DR, Barnett MJ, et al. A randomized multicenter comparison of bone marrow and peripheral blood in recipients of matched sibling allogeneic transplants for myeloid malignancies. Blood 2002:100(5):1525-1531.

181. Jennings MT, Sposto R, Boyett JM, et al. Preradiation chemotherapy in primary high-risk brainstem tumors: phase II study CCG-9941 of the Children's Cancer Group. Journal of Clinical Oncology 2002:20(16):3431-3437.

182. Kamps WA, Bokkerink JPM, HakvoortCammel FGAJ, et al. BFM-oriented treatment for children with acute lymphoblastic leukemia without cranial irradiation and treatment reduction for standard risk patients: Results of DCLSG protocol ALL-8 (1991-1996). Leukemia 2002:16(6):1099-1111.

183. Katzenstein HM, Krailo MD, Malogolowkin MH, et al. Hepatocellular carcinoma in children and adolescents: results from the Pediatric Oncology Group and the Children's Cancer Group intergroup study. Journal of Clinical Oncology 2002:20(12):2789-2797.

184. Nachman JB, Sposto R, Herzog P, et al. Randomized comparison of low-dose involved-field radiotherapy and no radiotherapy for children with Hodgkin's disease who achieve a complete response to chemotherapy. Journal of Clinical Oncology 2002:20(18):3765-3771.

185. Perel Y, Auvrignon A, Leblanc T, et al. Impact of addition of maintenance therapy to intensive induction and consolidation chemotherapy for childhood acute myeloblastic leukemia: Results of a prospective randomized trial, LAME 89/91. Journal of Clinical Oncology 2002:20(12):2774-2782.

186. Woods WG, Barnard DR, Alonzo TA, et al. Prospective study of 90 children requiring treatment for juvenile myelomonocytic leukemia or myelodysplastic syndrome: A report from the Children's Cancer Group. Journal of Clinical Oncology 2002:20(2):434-440.

187. Bertolone SJ, Yates AJ, Boyett JM, et al. Combined modality therapy for poorly differentiated gliomas of the posterior fossa in children: a Children's Cancer Group report. Journal of neuro-oncology 2003:63(1):49-54.

188. Boissel N, Auclerc MF, Lheritier V, et al. Should adolescents with acute lymphoblastic leukemia be treated as old children or young adults? Comparison of the French FRALLE-93 and LALA-94 trials. Journal of clinical oncology : official journal of the American Society of Clinical Oncology 2003:21(5):774-780.

189. Bunin N, Aplenc R, Kamani N, et al. Randomized trial of busulfan vs total body irradiation containing conditioning regimens for children with acute lymphoblastic leukemia: A pediatric blood and marrow transplant consortium study. Bone marrow transplantation 2003:32(6):543-548.

190. Cairo MS, Sposto R, HooverRegan M, et al. Childhood and adolescent large-cell lymphoma (LCL): A review of the Children's Cancer Group experience. American Journal of Hematology 2003:72(1):53-63.

191. Clarke M, Gaynon P, Hann I, et al. CNS-directed therapy for childhood acute lymphoblastic leukemia: Childhood ALL Collaborative Group overview of 43 randomized trials. Journal of clinical oncology : official journal of the American Society of Clinical Oncology 2003:21(9):1798-1809.

192. Goorin AM, Schwartzentruber DJ, Devidas M, et al. Presurgical chemotherapy compared with immediate surgery and adjuvant chemotherapy for nonmetastatic osteosarcoma: Pediatric Oncology Group Study POG-8651. Journal of Clinical Oncology 2003:21(8):1574-1580.

193. Haas-Kogan DA, Swift PS, Selch M, et al. Impact of radiotherapy for high-risk neuroblastoma: a Children's Cancer Group study. International journal of radiation oncology, biology, physics 2003:56(1):28-39.

194. Heath JA, Steinherz PG, Altman A, et al. Human granulocyte colony-stimulating factor in children with high-risk acute lymphoblastic leukemia: a Children's Cancer Group Study. Journal of Clinical Oncology 2003:21(8):1612-1617.

195. Hutchinson RJ, Gaynon PS, Sather H, et al. Intensification of therapy for children with lower-risk acute lymphoblastic leukemia: long-term follow-up of patients treated on Children's Cancer Group Trial 1881. Journal of Clinical Oncology 2003:21(9):1790-1797.

196. Katzenstein HM, Krailo MD, Malogolowkin MH, et al. Fibrolamellar hepatocellular carcinoma in children and adolescents. Cancer 2003:97(8):2006-2012.

197. Schmiegelow K, Bjork O, Glomstein A, et al. Intensification of mercaptopurine/methotrexate maintenance chemotherapy may increase the risk of relapse for some children with acute lymphoblastic leukemia. Journal of Clinical Oncology 2003:21(7):1332-1339.

198. Taylor RE, Bailey CC, Robinson K, et al. Results of a randomized study of preradiation chemotherapy versus radiotherapy alone for nonmetastatic medulloblastoma: The International Society of Paediatric Oncology/United Kingdom Children's Cancer Study Group PNET-3 Study. Journal of Clinical Oncology 2003:21(8):1581-1591.

199. Yetgin S, Tuncer MA, Cetin M, et al. Benefit of high-dose methylprednisolone in comparison with conventional-dose prednisolone during remission induction therapy in childhood acute lymphoblastic leukemia for long-term follow-up. Leukemia 2003:17(2):328-333.

200. Cushing B, Giller R, Cullen JW, et al. Randomized comparison of combination chemotherapy with etoposide, bleomycin, and either high-dose or standard-dose cisplatin in children and adolescents with high-risk malignant germ cell tumors: a pediatric intergroup study--Pediatric Oncology Group 9049 and Children's Cancer Group 8882. Journal of Clinical Oncology 2004:22(13):2691-2700.

201. Laskar S, Gupta T, Vimal S, et al. Consolidation radiation after complete remission in Hodgkin's disease following six cycles of doxorubicin, bleomycin, vinblastine, and dacarbazine chemotherapy: is there a need? J Clin Oncol 2004:22(1):62-68.

202. Lehrnbecher T, Varwig D, Kaiser J, et al. Infectious complications in pediatric acute myeloid leukemia: Analysis of the prospective multi-institutional clinical trial AML-BFM 93. Leukemia 2004:18(1):72-77.

203. LopezHernandez MA, Alvarado M, De Diego J, et al. A randomized trial of dexamethasone before remission induction, in de novo childhood acute lymphoblastic leukemia. Haematologica 2004:89(3):365-366.

204. Milpied N, Deconinck E, Gaillard F, et al. Initial Treatment of Aggressive Lymphoma with High-Dose Chemotherapy and Autologous Stem-Cell Support. New England Journal of Medicine 2004:350(13):1287-1295.

205. Miser JS, Krailo MD, Tarbell NJ, et al. Treatment of metastatic Ewing's sarcoma or primitive neuroectodermal tumor of bone: evaluation of combination ifosfamide and etoposide--a Children's Cancer Group and Pediatric Oncology Group study. Journal of Clinical Oncology 2004:22(14):2873-2876.

206. Neudorf S, Sanders J, Kobrinsky N, et al. Allogeneic bone marrow transplantation for children with acute myelocytic leukemia in first remission demonstrates a role for graft versus leukemia in the maintenance of disease-free survival. Blood 2004:103(10):3655-3661.

207. Reinhard H, Semler O, Burger D, et al. Results of the SIOP 93-01/GPOH trial and study for the treatment of patients with unilateral nonmetastatic wilms tumor. Klinische Padiatrie 2004:216(3):132-140.

208. Straus DJ, Portlock CS, Qin J, et al. Results of a prospective randomized clinical trial of doxorubicin, bleomycin, vinblastine, and dacarbazine (ABVD) followed by radiation therapy (RT) versus ABVD alone for stages I, II, and IIIA nonbulky Hodgkin disease. Blood 2004:104(12):3483-3489.

209. Suh C, Kim HJ, Kim SH, et al. Low-dose lenograstim to enhance engraftment after autologous stem cell transplantation: A prospective randomized evaluation of two different fixed doses. Transfusion 2004:44(4):533-538.

210. Waber DP, Silverman LB, Catania L, et al. Outcomes of a randomized trial of hyperfractionated cranial radiation therapy for treatment of high-risk acute lymphoblastic leukemia: Therapeutic efficacy and neurotoxicity. Journal of Clinical Oncology 2004:22(13):2701-2707.

211. Einsiedel HG, von Stackelberg A, Hartmann R, et al. Long-term outcome in children with relapsed ALL by risk-stratified salvage therapy: results of trial acute lymphoblastic leukemia-relapse study of the Berlin-Frankfurt-Munster Group 87. Journal of clinical oncology : official journal of the American Society of Clinical Oncology 2005:23(31):7942-7950.

212. George RE, London WB, Cohn SL, et al. Hyperdiploidy plus nonamplified MYCN confers a favorable prognosis in children 12 to 18 months old with disseminated neuroblastoma: a Pediatric Oncology Group study. Journal of clinical oncology : official journal of the American Society of Clinical Oncology 2005:23(27):6466-6473.

213. Gibson BES, Wheatley K, Hann IM, et al. Treatment strategy and long-term results in paediatric patients treated in consecutive UK AML trials. Leukemia 2005:19(12):2130-2138.

214. Igarashi S, Manabe A, Ohara A, et al. No advantage of dexamethasone over prednisolone for the outcome of standard- and intermediate-risk childhood acute lymphoblastic leukemia in the Tokyo Children's Cancer Study Group L95-14 protocol. Journal of Clinical Oncology 2005:23(27):6489-6498.

215. Laver JH, Kraveka JM, Hutchison RE, et al. Advanced-stage large-cell lymphoma in children and adolescents: results of a randomized trial incorporating intermediate-dose methotrexate and high-dose cytarabine in the maintenance phase of the APO regimen: a Pediatric Oncology Group phase III trial. Journal of Clinical Oncology 2005:23(3):541-547.

216. MacDonald TJ, Arenson EB, Ater J, et al. Phase II study of high-dose chemotherapy before radiation in children with newly diagnosed high-grade astrocytoma: Final Analysis of Children's Cancer Group Study 9933. Cancer 2005:104(12):2862-2871.

217. Meyers PA, Schwartz CL, Krailo M, et al. Osteosarcoma: A randomized, prospective trial of the addition of ifosfamide and/or muramyl tripeptide to cisplatin, doxorubicin, and high-dose methotrexate. Journal of Clinical Oncology 2005:23(9):2004-2011.

218. Pession A, Valsecchi MG, Masera G, et al. Long-term results of a randomized trial on extended use of high dose L-asparaginase for standard risk childhood acute lymphoblastic leukemia. Journal of Clinical Oncology 2005:23(28):7161-7167.

219. Smith FO, Alonzo TA, Gerbing RB, et al. Long-term results of children with acute myeloid leukemia: a report of three consecutive Phase III trials by the Children's Cancer Group: CCG 251, CCG 213 and CCG 2891. Leukemia 2005:19(12):2054-2062.

220. Stevens MC, Rey A, Bouvet N, et al. Treatment of nonmetastatic rhabdomyosarcoma in childhood and adolescence: third study of the International Society of Paediatric Oncology--SIOP Malignant Mesenchymal Tumor 89. Journal of clinical oncology : official journal of the American Society of Clinical Oncology 2005:23(12):2618-2628.

221. Testi AM, Biondi A, Lo Coco F, et al. GIMEMA-AIEOP AIDA protocol for the treatment of newly diagnosed acute promyelocytic leukemia (APL) in children. Blood 2005:106(2):447-453.

222. Tolar J, Bostrom BC, La MK, et al. Intravenous 6-mercaptopurine decreases salvage after relapse in childhood acute lymphoblastic leukemia: a report from the Children's Cancer Group study CCG 1922. Pediatric Blood & Cancer 2005:45(1):5-9.

223. Woessmann W, Seidemann K, Mann G, et al. The impact of the methotrexate administration schedule and dose in the treatment of children and adolescents with B-cell neoplasms: a report of the BFM Group Study NHL-BFM95. Blood 2005:105(3):948-958.

224. Bernstein ML, Devidas M, Lafreniere D, et al. Intensive therapy with growth factor support for patients with Ewing tumor metastatic at diagnosis: Pediatric Oncology Group/Children's Cancer Group Phase II Study 9457--a report from the Children's Oncology Group. Journal of Clinical Oncology 2006:24(1):152-159.

225. Mahoney Jr DH, Camitta BM, Devidas M. Does intravenous 6-mercaptopurine decrease salvage after relapse in childhood acute lymphoblastic leukemia? [3]. Pediatric Blood and Cancer 2006:46(5):660-661.

226. Malogolowkin MH, Katzenstein H, Krailo MD, et al. Intensified platinum therapy is an ineffective strategy for improving outcome in pediatric patients with advanced hepatoblastoma. Journal of Clinical Oncology 2006:24(18):2879-2884.

227. Pollack IF, Hamilton RL, Sobol RW, et al. O6-Methylguanine-DNA methyltransferase expression strongly correlates with outcome in childhood malignant gliomas: Results from the CCG-945 cohort. Journal of Clinical Oncology 2006:24(21):3431-3437.

228. Adamson PC, Matthay KK, O'Brien M, et al. A phase 2 trial of all-trans-retinoic acid in combination with interferon-alpha2a in children with recurrent neuroblastoma or wilms tumor: A pediatric oncology branch, NCI and children's oncology group study. Pediatric Blood and Cancer 2007:49(5):661-665.

229. Bhatia S, Krailo MD, Chen Z, et al. Therapy-related myelodysplasia and acute myeloid leukemia after Ewing sarcoma and primitive neuroectodermal tumor of bone: A report from the Children's Oncology Group. Blood 2007:109(1):46-51.

230. Cairo MS, Gerrard M, Sposto R, et al. Results of a randomized international study of high-risk central nervous system B non-Hodgkin lymphoma and B acute lymphoblastic leukemia in children and adolescents. Blood 2007:109(7):2736-2743.

231. Dinndorf PA, Gootenberg J, Cohen MH, et al. FDA drug approval summary: Pegaspargase (Oncaspar) for the first-line treatment of children with acute lymphoblastic leukemia (ALL). Oncologist 2007:12(8):991-998.

232. Lehrnbecher T, Zimmermann M, Reinhardt D, et al. Prophylactic human granulocyte colony-stimulating factor after induction therapy in pediatric acute myeloid leukemia. Blood 2007:109(3):936-943.

233. Lewis IJ, Nooij MA, Whelan J, et al. Improvement in histologic response but not survival in osteosarcoma patients treated with intensified chemotherapy: A randomized phase III trial of the european osteosarcoma intergroup. Journal of the National Cancer Institute 2007:99(2):112-128.

234. Moghrabi A, Levy DE, Asselin B, et al. Results of the Dana-Farber Cancer Institute ALL Consortium Protocol 95-01 for children with acute lymphoblastic leukemia. Blood 2007:109(3):896-904.

235. Neudorf S, Sanders J, Kobrinsky N, et al. Autologous bone marrow transplantation for children with AML in first remission. Bone marrow transplantation 2007:40(4):313-318.

236. Pieters R, Schrappe M, De Lorenzo P, et al. A treatment protocol for infants younger than 1 year with acute lymphoblastic leukaemia (Interfant-99): an observational study and a multicentre randomised trial. Lancet 2007:370(9583):240-250.

237. Ribera JM, Ortega JJ, Oriol A, et al. Comparison of intensive chemotherapy, allogeneic, or autologous stem-cell transplantation as postremission treatment for children with very high risk acute lymphoblastic leukemia: PETHEMA ALL-93 trial. Journal of Clinical Oncology 2007:25(1):16-24.

238. Rutkowski S, von Bueren A, von Hoff K, et al. Prognostic relevance of clinical and biological risk factors in childhood medulloblastoma: results of patients treated in the prospective multicenter trial HIT'91. Clinical Cancer Research 2007:13(9):2651-2657.

239. Skapek SX, Ferguson WS, Granowetter L, et al. Vinblastine and methotrexate for desmoid fibromatosis in children: Results of a Pediatric Oncology Group phase II trial. Journal of Clinical Oncology 2007:25(5):501-506.

240. Tebbi CK, London WB, Friedman D, et al. Dexrazoxane-associated risk for acute myeloid leukemia/myelodysplastic syndrome and other secondary malignancies in pediatric Hodgkin's disease. Journal of Clinical Oncology 2007:25(5):493-500.

241. Arico M, Valsecchi MG, Rizzari C, et al. Long-term results of the AIEOP-ALL-95 trial for childhood acute lymphoblastic leukemia: Insight on the prognostic value of DNA index in the framework of Berlin-Frankfurt-Muenster-based chemotherapy. Journal of Clinical Oncology 2008:26(2):283-289.

242. Barry EV, Vrooman LM, Dahlberg SE, et al. Absence of secondary malignant neoplasms in children with high-risk acute lymphoblastic leukemia treated with dexrazoxane. Journal of Clinical Oncology 2008:26(7):1106-1111.

243. Bhatla D, Gerbing RB, Alonzo TA, et al. DNA repair polymorphisms and outcome of chemotherapy for acute myelogenous leukemia: A report from the Children's Oncology Group. Leukemia 2008:22(2):265-272.

244. Bond M, Bernstein ML, Pappo A, et al. A phase II study of imatinib mesylate in children with refractory or relapsed solid tumors: A children's oncology group study. Pediatric Blood and Cancer 2008:50(2):254-258.

245. Bradley KA, Pollack IF, Reid JM, et al. Motexafin gadolinium and involved field radiation therapy for intrinsic pontine glioma of childhood: A Children's Oncology Group phase i study. Neuro-oncology 2008:10(5):752-758.

246. Gadner H, Grois N, Potschger U, et al. Improved outcome in multisystem Langerhans cell histiocytosis is associated with therapy intensification. Blood 2008:111(5):2556-2562.

247. Gangopadhyay AN, Rajeev R, Sharma SP, et al. Anterior intratumoural chemotherapy: a newer modality of treatment in advanced solid tumours in children. Asian Journal of Surgery 2008:31(4):225-229.

248. Lange BJ, Smith FO, Feusner J, et al. Outcomes in CCG-2961, a children's oncology group phase 3 trial for untreated pediatric acute myeloid leukemia: a report from the children's oncology group. Blood 2008:111(3):1044-1053.

249. Meyers PA, Schwartz CL, Krailo MD, et al. Osteosarcoma: the addition of muramyl tripeptide to chemotherapy improves overall survival--a report from the Children's Oncology Group. Journal of Clinical Oncology 2008:26(4):633-638.

250. Moricke A, Reiter A, Zimmermann M, et al. Risk-adjusted therapy of acute lymphoblastic leukemia can decrease treatment burden and improve survival: treatment results of 2169 unselected pediatric and adolescent patients enrolled in the trial ALL-BFM 95. Blood 2008:111(9):4477-4489.

251. Paulussen M, Craft AW, Lewis I, et al. Results of the EICESS-92 study: Two randomized trials of Ewing's sarcoma treatment - Cyclophosphamide compared with ifosfamide in standard-risk patients and assessment of benefit of etoposide added to standard treatment in high-risk patients. Journal of Clinical Oncology 2008:26(27):4385-4393.

252. Seibel NL, Steinherz PG, Sather HN, et al. Early postinduction intensification therapy improves survival for children and adolescents with high-risk acute lymphoblastic leukemia: a report from the Children's Oncology Group. Blood 2008:111(5):2548-2555.

253. Bhatla D, Gerbing RB, Alonzo TA, et al. Cytidine deaminase genotype and toxicity of cytosine arabinoside therapy in children with acute myeloid leukemia. British journal of haematology 2009:144(3):388-394.

254. Brugieres L, Le Deley M-C, Rosolen A, et al. Impact of the methotrexate administration dose on the need for intrathecal treatment in children and adolescents with anaplastic large-cell lymphoma: results of a randomized trial of the EICNHL Group. Journal of Clinical Oncology 2009:27(6):897-903.

255. Chou AJ, Kleinerman ES, Krailo MD, et al. Addition of muramyl tripeptide to chemotherapy for patients with newly diagnosed metastatic osteosarcoma: a report from the Children's Oncology Group. Cancer 2009:115(22):5339-5348.

256. Granowetter L, Womer R, Devidas M, et al. Dose-intensified compared with standard chemotherapy for nonmetastatic Ewing sarcoma family of tumors: a Children's Oncology Group Study. Journal of Clinical Oncology 2009:27(15):2536-2541.

257. Lanino E, Rondelli R, Locatelli F, et al. Early (day -7) versus conventional (day -1) inception of cyclosporine-A for graft-versus-host disease prophylaxis after unrelated donor hematopoietic stem cell transplantation in children. Long-term results of an AIEOP prospective, randomized study. Biology of blood and marrow transplantation : journal of the American Society for Blood and Marrow Transplantation 2009:15(6):741-748.

258. Matthay KK, Reynolds CP, Seeger RC, et al. Long-term results for children with high-risk neuroblastoma treated on a randomized trial of myeloablative therapy followed by 13-cis-retinoic acid: a children's oncology group study. Journal of Clinical Oncology 2009:27(7):1007-1013.

259. Nachman JB, La MK, Hunger SP, et al. Young adults with acute lymphoblastic leukemia have an excellent outcome with chemotherapy alone and benefit from intensive postinduction treatment: a report from the children's oncology group. Journal of Clinical Oncology 2009:27(31):5189-5194.

260. Rubnitz JE, Crews KR, Pounds S, et al. Combination of cladribine and cytarabine is effective for childhood acute myeloid leukemia: Results of the St Jude AML97 trial. Leukemia 2009:23(8):1410-1416.

261. von Hoff K, Hinkes B, Gerber NU, et al. Long-term outcome and clinical prognostic factors in children with medulloblastoma treated in the prospective randomised multicentre trial HIT'91. European journal of cancer 2009:45(7):1209-1217.

262. De Moerloose B, Suciu S, Bertrand Y, et al. Improved outcome with pulses of vincristine and corticosteroids in continuation therapy of children with average risk acute lymphoblastic leukemia (ALL) and lymphoblastic non-Hodgkin lymphoma (NHL): report of the EORTC randomized phase 3 trial 58951. Blood 2010:116(1):36-44.

263. Ehlers S, Herbst C, Zimmermann M, et al. Granulocyte colony-stimulating factor (G-CSF) treatment of childhood acute myeloid leukemias that overexpress the differentiation-defective G-CSF receptor isoform IV is associated with a higher incidence of relapse. Journal of Clinical Oncology 2010:28(15):2591-2597.

264. Johnson PWM, Sydes MR, Hancock BW, et al. Consolidation radiotherapy in patients with advanced Hodgkin's lymphoma: Survival data from the UKLG LY09 randomized controlled trial (ISRCTN97144519). Journal of Clinical Oncology 2010:28(20):3352-3359.

265. Liang DC, Yang CP, Lin DT, et al. Long-term results of Taiwan Pediatric Oncology Group studies 1997 and 2002 for childhood acute lymphoblastic leukemia. Leukemia 2010:24(2):397-405.

266. London WB, Frantz CN, Campbell LA, et al. Phase II randomized comparison of topotecan plus cyclophosphamide versus topotecan alone in children with recurrent or refractory neuroblastoma: a Children's Oncology Group study. Journal of Clinical Oncology 2010:28(24):3808-3815.

267. Mascarenhas L, Lyden ER, Breitfeld PP, et al. Randomized phase II window trial of two schedules of irinotecan with vincristine in patients with first relapse or progression of rhabdomyosarcoma: a report from the Children's Oncology Group. Journal of Clinical Oncology 2010:28(30):4658-4663.

268. Moorman AV, Ensor HM, Richards SM, et al. Prognostic effect of chromosomal abnormalities in childhood B-cell precursor acute lymphoblastic leukaemia: Results from the UK Medical Research Council ALL97/99 randomised trial. The Lancet Oncology 2010:11(5):429-438.

269. Parker C, Waters R, Leighton C, et al. Effect of mitoxantrone on outcome of children with first relapse of acute lymphoblastic leukaemia (ALL R3): an open-label randomised trial. Lancet 2010:376(9757):2009-2017.

270. Stork LC, Matloub Y, Broxson E, et al. Oral 6-mercaptopurine versus oral 6-thioguanine and veno-occlusive disease in children with standard-risk acute lymphoblastic leukemia: report of the Children's Oncology Group CCG-1952 clinical trial. Blood 2010:115(14):2740-2748.

271. Tallen G, Ratei R, Mann G, et al. Long-term outcome in children with relapsed acute lymphoblastic leukemia after time-point and site-of-relapse stratification and intensified short-course multidrug chemotherapy: Results of trial ALL-REZ BFM 90. Journal of Clinical Oncology 2010:28(14):2339-2347.

272. Tsuchida M, Ohara A, Manabe A, et al. Long-term results of Tokyo children's cancer study group trials for childhood acute lymphoblastic leukemia, 1984-1999. Leukemia 2010:24(2):383-396.

273. Vora AJ, Mitchell C, Goulden N, et al. UKALL 2003, a randomised trial investigating treatment reduction for children and young adults with minimal residual disease defined low risk acute lymphoblastic leukaemia. 52nd Annual Meeting of the American Society of Hematology, ASH 2010 Orlando, FL United States 2010:116 (21) (no pagination.

274. Yu AL, Gilman AL, Ozkaynak MF, et al. Anti-GD2 antibody with GM-CSF, interleukin-2, and isotretinoin for neuroblastoma. N Engl J Med 2010:363(14):1324-1334.

275. Asselin BL, Devidas M, Wang C, et al. Effectiveness of high-dose methotrexate in T-cell lymphoblastic leukemia and advanced-stage lymphoblastic lymphoma: a randomized study by the Children's Oncology Group (POG 9404). Blood 2011:118(4):874-883.

276. Freyer DR, Devidas M, La M, et al. Postrelapse survival in childhood acute lymphoblastic leukemia is independent of initial treatment intensity: A report from the Children's Oncology Group. Blood 2011:117(11):3010-3015.

277. Gibson BES, Webb DKH, Howman AJ, et al. Results of a randomized trial in children with Acute Myeloid Leukaemia: Medical Research Council AML12 trial. British journal of haematology 2011:155(3):366-376.

278. Horstmann M, Escherich G. Treatment of acute lymphoblastic leucemia of childhood: Interim report CoALL 08-09. 78 Wissenschaftlichen Halbjahrestagung der Gesellschaft fur Padiatrische Onkologie und Hamatologie, GPOH Frankfurt Germany 2011:159(10):1006-1007.

279. Kramm C, Roth D, Wolff JEA. First results of the randomized clinical trial HIT-GBM-D for treatment of children and adolescents with high grade glioma. 78 Wissenschaftlichen Halbjahrestagung der Gesellschaft fur Padiatrische Onkologie und Hamatologie, GPOH Frankfurt Germany 2011:159(10):1005.

280. Kurtzberg J, Asselin B, Bernstein M, et al. Polyethylene Glycol-conjugated L-asparaginase versus native L-asparaginase in combination with standard agents for children with acute lymphoblastic leukemia in second bone marrow relapse: a Children's Oncology Group Study (POG 8866). Journal of Pediatric Hematology/Oncology 2011:33(8):610-616.

281. Matloub Y, Bostrom BC, Hunger SP, et al. Escalating intravenous methotrexate improves event-free survival in children with standard-risk acute lymphoblastic leukemia: a report from the Children's Oncology Group. Blood 2011:118(2):243-251.

282. Von Bueren AO, Von Hoff K, Pietsch T, et al. Treatment of young children with localized medulloblastoma by chemotherapy alone: Results of the prospective, multicenter trial HIT 2000 confirming the prognostic impact of histology. Neuro-oncology 2011:13(6):669-679.

283. Vrooman LM, Neuberg DS, Stevenson KE, et al. The low incidence of secondary acute myelogenous leukaemia in children and adolescents treated with dexrazoxane for acute lymphoblastic leukaemia: a report from the Dana-Farber Cancer Institute ALL Consortium. European journal of cancer 2011:47(9):1373-1379.

284. Biondi A, Schrappe M, De Lorenzo P, et al. Imatinib after induction for treatment of children and adolescents with Philadelphia-chromosome-positive acute lymphoblastic leukaemia (EsPhALL): a randomised, open-label, intergroup study. Lancet Oncology 2012:13(9):936-945.

285. Lannering B, Rutkowski S, Doz F, et al. Hyperfractionated versus conventional radiotherapy followed by chemotherapy in standard-risk medulloblastoma: Results from the randomized multicenter HIT-SIOP PNET 4 trial. Journal of Clinical Oncology 2012:30(26):3187-3193.

286. Lipshultz SE, Miller TL, Lipsitz SR, et al. Continuous Versus Bolus Infusion of Doxorubicin in Children With ALL: Long-term Cardiac Outcomes. Pediatrics 2012:130(6):1003-1011.

287. Tebbi CK, Mendenhall NP, London WB, et al. Response-dependent and reduced treatment in lower risk Hodgkin lymphoma in children and adolescents, results of P9426: a report from the Children's Oncology Group. Pediatric Blood & Cancer 2012:59(7):1259-1265.

288. Vu K, Busaidy N, Cabanillas ME, et al. A randomized controlled trial of an intensive insulin regimen in patients with hyperglycemic acute lymphoblastic leukemia. Clinical Lymphoma, Myeloma and Leukemia 2012:12(5):355-362.

289. Wolden SL, Chen L, Kelly KM, et al. Long-term results of CCG 5942: a randomized comparison of chemotherapy with and without radiotherapy for children with Hodgkin's lymphoma--a report from the Children's Oncology Group. Journal of Clinical Oncology 2012:30(26):3174-3180.

290. Womer RB, West DC, Krailo MD, et al. Randomized controlled trial of interval-compressed chemotherapy for the treatment of localized Ewing sarcoma: a report from the Children's Oncology Group. Journal of Clinical Oncology 2012:30(33):4148-4154.

291. Escherich G, Zimmermann M, JankaSchaub G. Doxorubicin or daunorubicin given upfront in a therapeutic window are equally effective in children with newly diagnosed acute lymphoblastic leukemia. A randomized comparison in trial CoALL 07-03. Pediatric Blood and Cancer 2013:60(2):254-257.

292. Fouladi M, Stewart CF, Blaney SM, et al. A molecular biology and phase II trial of lapatinib in children with refractory CNS malignancies: a pediatric brain tumor consortium study. Journal of neuro-oncology 2013:114(2):173-179.

293. Heerema NA, Carroll AJ, Devidas M, et al. Intrachromosomal amplification of chromosome 21 is associated with inferior outcomes in children with acute lymphoblastic leukemia treated in contemporary standard-risk children's oncology group studies: a report from the children's oncology group. Journal of clinical oncology : official journal of the American Society of Clinical Oncology 2013:31(27):3397-3402.

294. Packer RJ, Zhou T, Holmes E, et al. Survival and secondary tumors in children with medulloblastoma receiving radiotherapy and adjuvant chemotherapy: results of Children's Oncology Group trial A9961. Neuro-oncology 2013:15(1):97-103.

295. Vose JM, Carter S, Burns LJ, et al. Phase III randomized study of rituximab/carmustine, etoposide, cytarabine, and melphalan (BEAM) compared with iodine-131 tositumomab/BEAM with autologous hematopoietic cell transplantation for relapsed diffuse large B-cell lymphoma: results from the BMT CTN 0401 trial. Journal of clinical oncology : official journal of the American Society of Clinical Oncology 2013:31(13):1662-1668.

296. Vrooman LM, Stevenson KE, Supko JG, et al. Postinduction dexamethasone and individualized dosing of Escherichia Coli L-asparaginase each improve outcome of children and adolescents with newly diagnosed acute lymphoblastic leukemia: results from a randomized study--Dana-Farber Cancer Institute ALL Consortium Protocol 00-01. Journal of Clinical Oncology 2013:31(9):1202-1210.

297. Alexander S, Kraveka JM, Weitzman S, et al. Advanced stage anaplastic large cell lymphoma in children and adolescents: results of ANHL0131, a randomized phase III trial of APO versus a modified regimen with vinblastine: a report from the children's oncology group. Pediatric Blood & Cancer 2014:61(12):2236-2242.

298. Attarbaschi A, Panzer-Grumayer R, Mann G, et al. Minimal residual disease-based treatment is adequate for relapse-prone childhood acute lymphoblastic leukemia with an intrachromosomal amplification of chromosome 21: the experience of the ALL-BFM 2000 trial. Klinische Padiatrie 2014:226(6-7):338-343.

299. Batra V, Sands SA, Holmes E, et al. Long-term survival of children less than six years of age enrolled on the ccg-945 phase iii trial for newly-diagnosed high-grade glioma: A report from the children's oncology group. Pediatric Blood and Cancer 2014:61(1):151-157.

300. Chagaluka G, Stanley C, Banda K, et al. Kaposi's sarcoma in children: an open randomised trial of vincristine, oral etoposide and a combination of vincristine and bleomycin. European journal of cancer 2014:50(8):1472-1481.

301. Creutzig U, Semmler J, Kaspers GL, et al. Re-induction with L-DNR/FLAG improves response after AML relapse, but not long-term survival. Klinische Padiatrie 2014:226(6-7):323-331.

302. Flaherty LE, Othus M, Atkins MB, et al. Southwest Oncology Group S0008: a phase III trial of high-dose interferon Alfa-2b versus cisplatin, vinblastine, and dacarbazine, plus interleukin-2 and interferon in patients with high-risk melanoma--an intergroup study of cancer and leukemia Group B, Children's Oncology Group, Eastern Cooperative Oncology Group, and Southwest Oncology Group. Journal of Clinical Oncology 2014:32(33):3771-3778.

303. Gamis AS, Alonzo TA, Meshinchi S, et al. Gemtuzumab ozogamicin in children and adolescents with de novo acute myeloid leukemia improves event-free survival by reducing relapse risk: results from the randomized phase III Children's Oncology Group trial AAML0531. Journal of Clinical Oncology 2014:32(27):3021-3032.

304. Kato M, Koh K, Manabe A, et al. No impact of high-dose cytarabine and asparaginase as early intensification with intermediate-risk paediatric acute lymphoblastic leukaemia: results of randomized trial TCCSG study L99-15. British journal of haematology 2014:164(3):376-383.

305. Mo XD, Zhao XY, Liu DH, et al. Umbilical cord blood transplantation and unmanipulated haploidentical hematopoietic SCT for pediatric hematologic malignances. Bone marrow transplantation 2014:49(8):1070-1075.

306. Mori T, Fukano R, Saito A, et al. Analysis of Japanese registration from the randomized international trial for childhood anaplastic large cell lymphoma (ALCL99-R1). [Rinsho ketsueki] The Japanese journal of clinical hematology 2014:55(5):526-533.

307. Pulsipher MA, Langholz B, Wall DA, et al. The addition of sirolimus to tacrolimus/methotrexate GVHD prophylaxis in children with ALL: a phase 3 Children's Oncology Group/Pediatric Blood and Marrow Transplant Consortium trial. Blood 2014:123(13):2017-2025.

308. Raemaekers JM, Andre MP, Federico M, et al. Omitting radiotherapy in early positron emission tomography-negative stage I/II Hodgkin lymphoma is associated with an increased risk of early relapse: Clinical results of the preplanned interim analysis of the randomized EORTC/LYSA/FIL H10 trial. Journal of clinical oncology : official journal of the American Society of Clinical Oncology 2014:32(12):1188-1194.

309. Roos DE, Smith JG. Randomized trial on radiotherapy for paediatric diffuse intrinsic pontine glioma (DIPG). Radiotherapy & Oncology 2014:113(3):425.

310. Shinagawa K, Yanada M, Sakura T, et al. Tamibarotene as maintenance therapy for acute promyelocytic leukemia: Results from a randomized controlled trial. Journal of Clinical Oncology 2014:32(33):3729-3735.

311. Stary J, Zimmermann M, Campbell M, et al. Intensive chemotherapy for childhood acute lymphoblastic leukemia: results of the randomized intercontinental trial ALL IC-BFM 2002. Journal of Clinical Oncology 2014:32(3):174-184.

312. Tower RL, Jones TL, Camitta BM, et al. Dose intensification of methotrexate and cytarabine during intensified continuation chemotherapy for high-risk B-precursor acute lymphoblastic leukemia: POG 9406: a report from the Children's Oncology Group. Journal of Pediatric Hematology/Oncology 2014:36(5):353-361.

313. Aly MMD, Hamza AF, Abdel Kader HM, et al. Therapeutic superiority of combined propranolol with short steroids course over propranolol monotherapy in infantile hemangioma. European journal of pediatrics 2015:174(11):1503-1509.

314. Borowitz MJ, Wood BL, Devidas M, et al. Prognostic significance of minimal residual disease in high risk B-ALL: a report from Children's Oncology Group study AALL0232. Blood 2015:126(8):964-971.

315. Chow EJ, Asselin BL, Schwartz CL, et al. Late Mortality After Dexrazoxane Treatment: A Report From the Children's Oncology Group. Journal of Clinical Oncology 2015:33(24):2639-2645.

316. Dharmarajan KV, Friedman DL, Schwartz CL, et al. Patterns of relapse from a phase 3 Study of response-based therapy for intermediate-risk Hodgkin lymphoma (AHOD0031): a report from the Children's Oncology Group. International journal of radiation oncology, biology, physics 2015:92(1):60-66.

317. Junjun J, Xuelian Z, Dhruba K, et al. Efficacy of preoperative chemotherapy in treatment of children with wilms' tumor: A meta-analysis. Iranian Journal of Pediatrics 2015:25(2) (pagination):Arte Number: e366. ate of Pubaton: 2015.

318. Karol SE, CoustanSmith E, Cao X, et al. Prognostic factors in children with acute myeloid leukaemia and excellent response to remission induction therapy. British journal of haematology 2015:168(1):94-101.

319. Ko RH, Jones TL, Radvinsky D, et al. Allergic reactions and antiasparaginase antibodies in children with high-risk acute lymphoblastic leukemia: A children's oncology group report. Cancer 2015:121(23):4205-4211.

320. O'Connor D, Bartram J, Enshaei A, et al. Integration of minimal residual disease with other patient risk factors identifies a population with very poor overall survival in pediatric ALL: Results from the UKALL 2003 trial. 57th Annual Meeting of the American Society of Hematology, ASH 2015 San Diego, CA United States 2015:126(23):1412.

321. Place AE, Stevenson KE, Vrooman LM, et al. Intravenous pegylated asparaginase versus intramuscular native Escherichia coli L-asparaginase in newly diagnosed childhood acute lymphoblastic leukaemia (DFCI 05-001): a randomised, open-label phase 3 trial. Lancet Oncology 2015:16(16):1677-1690.

322. Rodeberg DA, Wharam MD, Lyden ER, et al. Delayed primary excision with subsequent modification of radiotherapy dose for intermediate-risk rhabdomyosarcoma: A report from the Children's Oncology Group Soft Tissue Sarcoma Committee. International Journal of Cancer 2015:137(1):204-211.

323. Sellar RS, Rowntree C, Vora AJ, et al. Relapse in teenage and young adult (TYA) patients treated on a pediatric minimal residual disease (MRD) stratified protocol is associated with a poor outcome: Results from UKALL2003. 57th Annual Meeting of the American Society of Hematology, ASH 2015 San Diego, CA United States 2015:126(23):2493.

324. Winter SS, Dunsmore KP, Devidas M, et al. Safe integration of nelarabine into intensive chemotherapy in newly diagnosed T-cell acute lymphoblastic leukemia: Children's Oncology Group Study AALL0434. Pediatric Blood & Cancer 2015:62(7):1176-1183.

325. Wolden SL, Lyden ER, Arndt CA, et al. Local Control for Intermediate-Risk Rhabdomyosarcoma: Results From D9803 According to Histology, Group, Site, and Size: A Report From the Children's Oncology Group. International journal of radiation oncology, biology, physics 2015:93(5):1071-1076.

326. Asselin BL, Devidas M, Chen L, et al. Cardioprotection and safety of dexrazoxane in patients treated for newly diagnosed T-cell acute lymphoblastic leukemia or advanced-stage lymphoblastic non-Hodgkin lymphoma: A report of the Children's Oncology Group randomized trial Pediatric Oncology Group 9404. Journal of Clinical Oncology 2016:34(8):854-862.

327. Falsini B, Chiaretti A, Rizzo D, et al. Nerve growth factor improves visual loss in childhood optic gliomas: A randomized, double-blind, phase II clinical trial. Brain 2016:139(2):404-414.

328. Lucchese A, Matarese G, Manuelli M, et al. Reliability and efficacy of palifermin in prevention and management of oral mucositis in patients with acute lymphoblastic leukemia: A randomized, double-blind controlled clinical trial. Minerva stomatologica 2016:65(1):43-53.

329. Pollard JA, Loken M, Gerbing RB, et al. CD33 expression and its association with gemtuzumab ozogamicin response: Results from the randomized phase III children's oncology group trial AAML0531. Journal of Clinical Oncology 2016:34(7):747-755.

**Appendix C: PRISMA 2009 Checklist**

| **Section/topic** | **#** | **Checklist item** | **Reported on page #** |
| --- | --- | --- | --- |
| **TITLE** | | |  |
| Title | 1 | Identify the report as a systematic review, meta-analysis, or both. | 5 |
| **ABSTRACT** | | |  |
| Structured summary | 2 | Provide a structured summary including, as applicable: background; objectives; data sources; study eligibility criteria, participants, and interventions; study appraisal and synthesis methods; results; limitations; conclusions and implications of key findings; systematic review registration number. | 3-4 |
| **INTRODUCTION** | | |  |
| Rationale | 3 | Describe the rationale for the review in the context of what is already known. | 4-5 |
| Objectives | 4 | Provide an explicit statement of questions being addressed with reference to participants, interventions, comparisons, outcomes, and study design (PICOS). | Objectives page 4, but not appropriate for PICOS format |
| **METHODS** | | |  |
| Protocol and registration | 5 | Indicate if a review protocol exists, if and where it can be accessed (e.g., Web address), and, if available, provide registration information including registration number. | Developed but not published |
| Eligibility criteria | 6 | Specify study characteristics (e.g., PICOS, length of follow-up) and report characteristics (e.g., years considered, language, publication status) used as criteria for eligibility, giving rationale. | 5-6 |
| Information sources | 7 | Describe all information sources (e.g., databases with dates of coverage, contact with study authors to identify additional studies) in the search and date last searched. | 5 |
| Search | 8 | Present full electronic search strategy for at least one database, including any limits used, such that it could be repeated. | 5 & Appendix A |
| Study selection | 9 | State the process for selecting studies (i.e., screening, eligibility, included in systematic review, and, if applicable, included in the meta-analysis). | 6 |
| Data collection process | 10 | Describe method of data extraction from reports (e.g., piloted forms, independently, in duplicate) and any processes for obtaining and confirming data from investigators. | 6 |
| Data items | 11 | List and define all variables for which data were sought (e.g., PICOS, funding sources) and any assumptions and simplifications made. | Summarized in tables |
| Risk of bias in individual studies | 12 | Describe methods used for assessing risk of bias of individual studies (including specification of whether this was done at the study or outcome level), and how this information is to be used in any data synthesis. | N/A since we were assessing methodology and reporting rather than results |
| Summary measures | 13 | State the principal summary measures (e.g., risk ratio, difference in means). | 6-7 |
| Synthesis of results | 14 | Describe the methods of handling data and combining results of studies, if done, including measures of consistency (e.g., I^2^) for each meta-analysis. | 6/7 |

Page 1 of 2

| **Section/topic** | **#** | **Checklist item** | | **Reported on page #** |
| --- | --- | --- | --- | --- |
| Risk of bias across studies | 15 | Specify any assessment of risk of bias that may affect the cumulative evidence (e.g., publication bias, selective reporting within studies). | | N/A since we were assessing methodology and reporting rather than results |
| Additional analyses | 16 | Describe methods of additional analyses (e.g., sensitivity or subgroup analyses, meta-regression), if done, indicating which were pre-specified. | | 6-7 |
| **RESULTS** | | | |  |
| Study selection | | 17 | Give numbers of studies screened, assessed for eligibility, and included in the review, with reasons for exclusions at each stage, ideally with a flow diagram. | 7 (plus Figure 2) |
| Study characteristics | | 18 | For each study, present characteristics for which data were extracted (e.g., study size, PICOS, follow-up period) and provide the citations. | Table 1 |
| Risk of bias within studies | | 19 | Present data on risk of bias of each study and, if available, any outcome level assessment (see item 12). | N/A |
| Results of individual studies | | 20 | For all outcomes considered (benefits or harms), present, for each study: (a) simple summary data for each intervention group (b) effect estimates and confidence intervals, ideally with a forest plot. | N/A since we did not focus on outcome |
| Synthesis of results | | 21 | Present results of each meta-analysis done, including confidence intervals and measures of consistency. | N/A |
| Risk of bias across studies | | 22 | Present results of any assessment of risk of bias across studies (see Item 15). | N/A |
| Additional analysis | | 23 | Give results of additional analyses, if done (e.g., sensitivity or subgroup analyses, meta-regression [see Item 16]). | 8 (plus Tables 2, 3) |
| **DISCUSSION** | | | |  |
| Summary of evidence | | 24 | Summarize the main findings including the strength of evidence for each main outcome; consider their relevance to key groups (e.g., healthcare providers, users, and policy makers). | 8-13 |
| Limitations | | 25 | Discuss limitations at study and outcome level (e.g., risk of bias), and at review-level (e.g., incomplete retrieval of identified research, reporting bias). | 9 |
| Conclusions | | 26 | Provide a general interpretation of the results in the context of other evidence, and implications for future research. | 12-13 |
| **FUNDING** | | | |  |
| Funding | | 27 | Describe sources of funding for the systematic review and other support (e.g., supply of data); role of funders for the systematic review. | 13 |

*From:*  Moher D, Liberati A, Tetzlaff J, Altman DG, The PRISMA Group (2009). Preferred Reporting Items for Systematic Reviews and Meta-Analyses: The PRISMA Statement. PLoS Med 6(6): e1000097. doi:10.1371/journal.pmed1000097

For more information, visit: **www.prisma-statement.org**.

Page 2 of 2
